# Horizontal Gene Transfer to a Defensive Symbiont with a Reduced Genome in a Multipartite Beetle Microbiome

**DOI:** 10.1128/mBio.02430-19

**Published:** 2020-02-25

**Authors:** Samantha C. Waterworth, Laura V. Flórez, Evan R. Rees, Christian Hertweck, Martin Kaltenpoth, Jason C. Kwan

**Affiliations:** a Division of Pharmaceutical Sciences, School of Pharmacy, University of Wisconsin—Madison, Madison, Wisconsin, USA; b Department of Evolutionary Ecology, Institute of Organismic and Molecular Evolution, Johannes Gutenburg University, Mainz, Germany; c Department of Biomolecular Chemistry, Leibniz Institute for Natural Products Research and Infection Biology, Jena, Germany; d Department of Natural Product Chemistry, Friedrich Schiller University, Jena, Germany; McMaster University

**Keywords:** *Burkholderia*, insects, metagenomics, natural products, symbiosis

## Abstract

Symbiotic mutualisms of bacteria and animals are ubiquitous in nature, running a continuum from facultative to obligate from the perspectives of both partners. The loss of functions required for living independently but not within a host gives rise to reduced genomes in many symbionts. Although the phenomenon of genome reduction can be explained by existing evolutionary models, the initiation of the process is not well understood. Here, we describe the microbiome associated with the eggs of the beetle Lagria villosa, consisting of multiple bacterial symbionts related to Burkholderia gladioli, including a reduced-genome symbiont thought to be the exclusive producer of the defensive compound lagriamide. We show that the putative lagriamide-producing symbiont is the only member of the microbiome undergoing genome reduction and that it has already lost the majority of its primary metabolism and DNA repair pathways. The key step preceding genome reduction in the symbiont was likely the horizontal acquisition of the putative lagriamide *lga* biosynthetic gene cluster. Unexpectedly, we uncovered evidence of additional horizontal transfers to the symbiont’s genome while genome reduction was occurring and despite a current lack of genes needed for homologous recombination. These gene gains may have given the genome-reduced symbiont a selective advantage in the microbiome, especially given the maintenance of the large *lga* gene cluster despite ongoing genome reduction.

## Abstract (precis)

Associations between microorganisms and an animal, plant, or fungal host can result in increased dependence over time. This process is due partly to the bacterium not needing to produce nutrients that the host provides, leading to loss of genes that it would need to live independently and to a consequent reduction in genome size. It is often thought that genome reduction is aided by genetic isolation—bacteria that live in monocultures in special host organs, or inside host cells, have less access to other bacterial species from which they can obtain genes. Here, we describe exposure of a genome-reduced beetle symbiont to a community of related bacteria with nonreduced genomes. We show that the symbiont has acquired genes from other bacteria despite going through genome reduction, suggesting that isolation has not yet played a major role in this case of genome reduction, with horizontal gene gains still offering a potential route for adaptation.

## INTRODUCTION

Mutualistic symbioses between animals and bacteria, widespread in nature, serve a variety of functions such as biosynthesis of nutrients not found in the host’s diet (1, 2) and protection from predation (3–5) or infection (6–8). Such relationships exist on a continuous spectrum of dependency and exclusivity from the perspectives of both the host and the symbiont. Symbionts generally become obligate after a prolonged period of exclusive association with the host, and the symbionts that become obligate tend to carry out highly important functions for the host (9–11). For example, mitochondria and chloroplasts, organelles that are required for energy production and carbon fixation in eukaryotic and plant cells, originated from endosymbiotic capture of alphaproteobacteria and cyanobacteria ∼1.2 billion years ago (Bya) and ∼900 million years ago (Mya), respectively (12). The acquisition of these organelles allowed the diversification of eukaryotic species (13). More recently, aphids evolved to feed on plant sap depleted of several essential amino acids only through capture of an endosymbiont that can synthesize these nutrients, namely, Buchnera aphidicola, ∼160 to 280 Mya (14, 15). In these cases, the microbial symbiont has lost the ability to live independently of the host, and the hosts are also dependent on their symbionts.

The mechanism by which symbionts become obligate is through loss of genes required for independent (but not host-associated) life, leading to an overall reduction in genome size (9–11). This gene loss is the result of relaxed selection of genes for functions provided by the host and also of increased genetic drift as a consequence of the presence of small effective populations in strict vertical transmission (16, 17). A general mutational bias toward deletion in bacteria (18) combined with many successive population bottlenecks that allow the fixation of slightly deleterious mutations (10) mediates general gene degradation and genome reduction in symbionts. Also, a low level of recombination between different strains is often thought to contribute to this process. While these mechanisms are largely thought to be nonadaptive, there are also examples of free-living bacteria with reduced genomes, where reduction is thought to be the result of strong selection for reduced metabolic expenditure (19–21). In symbionts, there is also evidence that increases in AT content and reductions in genome size could be selected for to reduce the metabolic burden on the host (22, 23). The early stages of this process are manifested by a proliferation of nonfunctional pseudogenes and a decrease in coding density in the genome (11) before the intergenic sequences are lost to eventually give tiny (<1-Mbp) genomes (9, 10). While there is a robust model for the evolutionary forces that drive this process once a symbiont becomes host restricted, it is unknown how free-living bacteria first transition to an endosymbiotic lifestyle and start on the road to genome reduction (10).

The beetle subfamily Lagriinae offers an opportunity to examine this issue. Various Lagriinae species have evolved special symbiont-bearing structures that serve to facilitate the vertical transmission of bacteria (24). Symbionts are stored extracellularly in two accessory glands associated with the female reproductive system, allowing contamination of the egg surface with symbionts (24). These symbionts later colonize three compartments forming from invaginations of the dorsal cuticle in the larva (24). Beetles are typically coinfected with multiple symbiont strains related to the plant pathogen Burkholderia gladioli that are secreted onto the surface of eggs as they are laid (24). In the species Lagria villosa, a South American soybean pest, at least one symbiotic B. gladioli strain (Lv-StA) has been cultured and is still capable of infecting plants (24). The same strain produces antibacterial and antifungal compounds that can protect the beetle’s eggs from infection (24, 25). This is consistent with the hypothesis that the B. gladioli symbionts evolved from plant pathogens to become beetle mutualists. However, in field collections of L. villosa, Lv-StA is found only sporadically and is never highly abundant (26). Instead, the most abundant strain identified is often the uncultured Lv-StB strain (26), which we previously found harbors a biosynthetic gene cluster (BGC) predicted to produce lagriamide, a defensive compound found in field egg collections (6). We previously found through metagenomic sequencing that the genome of Lv-StB was much smaller than that of Lv-StA, suggesting that it has undergone genome reduction (6). It would seem that, while L. villosa has multiple options for symbionts that produce potential chemical defenses, only a subset have specialized as obligate mutualists. The presence of multiple related strains in this system, with selective genome reduction of a single strain, could potentially shed light on why the genomes of some symbionts become reduced.

Here, we show that in the L. villosa microbiome, Lv-StB is uniquely undergoing genome reduction, despite other community members possessing biosynthetic pathways for potentially defensive molecules. We also suggest that this process was likely driven not only by horizontal acquisition of the putative lagriamide pathway but also by loss of genes that limit cell division and translation and by gain of *zot*, encoding zonular occludens, a toxin also found in Vibrio cholerae that aids invasion of host membranes. Further, we present evidence that these horizontal gene transfers occurred concurrently with genome reduction, suggesting that complete genetic isolation is not a main driving force for the reduction process. (This article was submitted to an online preprint archive [27]).

## RESULTS AND DISCUSSION

### Selective genome reduction of strain Lv-StB in Lagria villosa.

We previously analyzed the metagenome of eight L. villosa egg clutches (6), using our binning pipeline Autometa (28). This method has the advantage that it can separate noncharacterized eukaryotic contamination from metagenomes, and it uses multiple factors (nucleotide composition, sequence homology, the presence of single-copy marker genes, and level of coverage) to accurately produce bins from individual data sets. Because we had implemented several bug fixes and small improvements to the pipeline since our original analysis, we re-ran Autometa on the same metagenomic assembly. Despite some minor differences, the new bins were broadly similar to our previous results (see Data Set S1A in the supplemental material), with 19 bins. As before, the Lv-StB bin had the highest coverage, at 1,977× (294 contigs; *N*_50_, 8,138 bp; see Data Set S1B for details of other bins), such that the constituent contigs are unlikely to represent repeats from lower-coverage bins. We classified the bins according to a new standardized bacterial taxonomy utilized by the Genome Taxonomy Database (GTDB) that minimizes polyphyletic taxa and standardizes divergence between taxa of the same rank (29). Notably, the GTDB taxonomy reclassifies betaproteobacteria as being under class *Gammaproteobacteria*. By this classification, all bins were in class *Gammaproteobacteria*, in three different orders: *Betaproteobacteriales*, *Pseudomonadales*, and *Xanthomonadales* (Data Set S1C). The most abundant bins were all in the family *Burkholderiaceae*, with the highest abundance corresponding to the Lv-StB strain previously found to harbor the putative lagriamide BGC (6). Interestingly, the average nucleotide identity (ANI) of Lv-StB to the reference B. gladioli genome in GTDB (strain ATCC 10248) is 85.7%, much lower than the 95% cutoff suggested for species identifications (30) (Data Set S1C). This divergence suggests that Lv-StB is a novel species in the genus *Burkholderia*, even though we previously classified it as B. gladioli on the basis of 16S rRNA gene sequence (6, 26), and therefore we refer to the strain here as “*Burkholderia* sp. Lv-StB.” Likewise, most bins were found to be novel species, with one (DBSCAN_round2_3) being divergent enough to be a representative of a novel genus in the family *Burkholderiaceae*. Notably, the cultured B. gladioli strain that we had previously isolated from L. villosa eggs, Lv-StA (6, 24) was not present in this metagenome.

10.1128/mBio.02430-19.6 DATA SET S1 Comparative analysis of the genome of *Burkholderia* sp. Lv-StB and other genomes in the genus *Burkholderia*. Download Data Set S1, XLSX file, 0.2 MB. Copyright © 2020 Waterworth et al. 2020 Waterworth et al. This content is distributed under the terms of the Creative Commons Attribution 4.0 International license.

**FIG 1:**
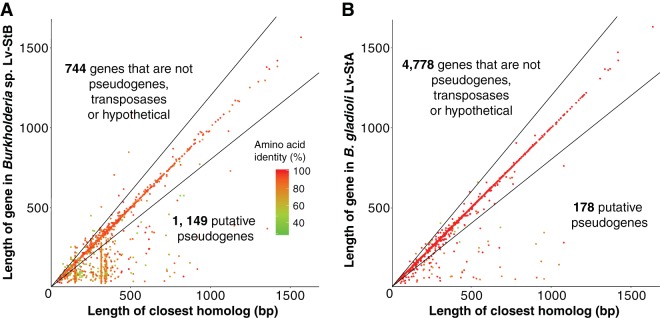
Comparison of the lengths of genes in the *Burkholderia* sp. Lv-StB genome (A) and B. gladioli Lv-StA (B) with the closest homologs identified through BLAST searches against the NR database (see Materials and Methods). Genes which are less than 80% of the length of the closest relative (i.e., below the lower black line) are putatively assigned as pseudogenes, as described previously (4, 121). Note that two vertical groupings of pseudogenes in Lv-StB correspond to multiple copies of a 155-bp hypothetical gene and an IS*5* family transposase.

The bins obtained had a range of different sizes (Table 1; see also Data Set S1B), which could be due to genome reduction or poor assembly and/or binning of a larger genome, which is often observed if there are many related strains in a metagenome (28). As part of the binning procedure, genome completeness was estimated based on the presence of 139 single-copy marker genes (31) (Data Set S1B). However, as some complete genomes of genome-reduced symbionts have low apparent completeness by this measure (32, 33), this figure cannot be used alone to determine the size of an incompletely assembled genome. Conversely, even the drastically reduced genomes of intracellular obligate insect symbionts have been found to almost universally maintain certain genes that we refer to here as “core genes,” involved in replication, transcription, protein folding/stability, tRNA modification, sulfur metabolism, RNA modification, and translation (9). We would expect, therefore, that a well-assembled reduced genome would contain a near-complete core gene set but not necessarily the whole set of 139 single-copy marker genes. Conversely, incompletely assembled genomes are likely to be missing a significant number of core genes that are required even in symbionts with highly reduced genomes. We examined the presence of core genes in all metagenomic bins, as well as in B. gladioli Lv-StA for comparison (Table 1; see also Data Set S1D). Nine bins were close in size to the genome of their respective closest relatives, while maintaining most core genes, and are classified as “nonreduced,” and 10 bins were small but also lacked a significant fraction of core genes and are classified as “incomplete.” Only the Lv-StB genome can be classified as reduced, on the basis of its reduced size compared to its close relative (2.07 Mbp, 23.5%) and maintenance of most core genes (85.7%). It is worth noting here that the “core” genes missing from the Lv-StB genome include genes essential for translation (*infB*, *trpS*, the tRNA-Met gene, and tRNA-Gln gene; Data Set S1D), which is probably explained by incomplete assembly of the draft-quality genome. The high level of maintenance of core genes nevertheless suggests that the assembly is missing a relatively small amount of the genome and that this genome is reduced in size. The Lv-StB bin exhibited additional features of genomes undergoing reduction, namely, reduced GC% content compared to the B. gladioli reference genome (58.7% versus 67.9%) and a proliferation of pseudogenes accounting for 45.29% of the annotated open reading frames (ORFs) (Fig. 1; see also Data Set S1C and E). Because of this, the Lv-StB genome exhibits low coding density (59.04%), and it also possesses a large number (*n* = 159) of ORFs containing transposases. Both of these characteristics are hallmarks of symbionts in the early stages of genome reduction, whose genomes contain high numbers of pseudogenes and genome rearrangements (9). The Lv-StB genome also contains a low number of genes compared with its free-living relative, with 744 ORFs that are not pseudogenes, transposase genes, or hypothetical genes versus 4,778 such genes in B. gladioli Lv-StA.

**TABLE 1:** Genome characteristics of *Burkholderia* symbiont Lv-StB, its relative *B. gladioli* Lv-StA, and other bins obtained from the metagenome

Genome	Coverage (×)	Size (% of closest relative)^*a*^	% of core genes	Category
B. gladioli Lv-StA	NA^*c*^	96.2	95.2	Nonreduced
B. gladioli sp. Lv-StB	1,977	23.5	85.7	Reduced
DBSCAN_cluster_round5_1	355	105	92.9	Nonreduced
DBSCAN_cluster_round2_3	298	74.7	95.2	Nonreduced
DBSCAN_cluster_round6_18	207	18.2	36.9	Incomplete
DBSCAN_cluster_round4_6	170	86.1	95.2	Nonreduced
DBSCAN_cluster_round43_4	142	4.74^*b*^	38.1	Incomplete
DBSCAN_cluster_round8_1	94	39.6	25	Incomplete
DBSCAN_cluster_round3_0	90	86.1	81	Nonreduced
DBSCAN_cluster_round5_3	71	77.2	81	Nonreduced
DBSCAN_cluster_round1_2	68	128	92.9	Nonreduced
DBSCAN_cluster_round6_14	61	20.9	39.3	Incomplete
DBSCAN_cluster_round66_39	47	0.351^*b*^	25	Incomplete
DBSCAN_cluster_round7_14	37	5.3	16.7	Incomplete
DBSCAN_cluster_round34_1	34	40.9	20.2	Incomplete
DBSCAN_cluster_round7_0	24	62.8	31	Incomplete
DBSCAN_cluster_round44_0	18	58.4	34.5	Incomplete
DBSCAN_cluster_round1_3	16	103	94	Nonreduced
DBSCAN_cluster_round4_0	8	102	89.3	Nonreduced
DBSCAN_cluster_round4_12	5	63.1	47.6	Incomplete

a Calculated relative to the genome of the closest relative identified by GTDB-Tk (see Data Set S1B).

b Calculated relative to the average size of 517 *Pseudomonas* genomes taken from the GTDB database.

c NA, not applicable.

### Diversity of biosynthetic gene clusters in the L. villosa microbiome.

**FIG 2:**
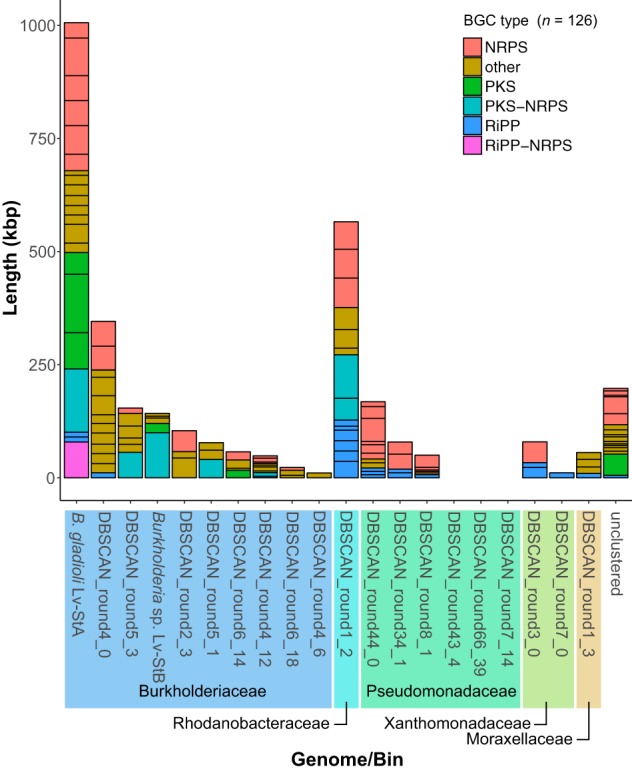
Distribution of biosynthetic gene clusters (BGCs) among the L. villosa metagenome bins and the genome of B. gladioli Lv-StA. Colors indicate the type of BGC annotated by antiSMASH, simplified into polyketide synthase (PKS), nonribosomal peptide synthetase (NRPS), ribosomally synthesized and posttranslationally modified peptides (RiPP), and “other” (126 identified) (34).

**FIG 3:**
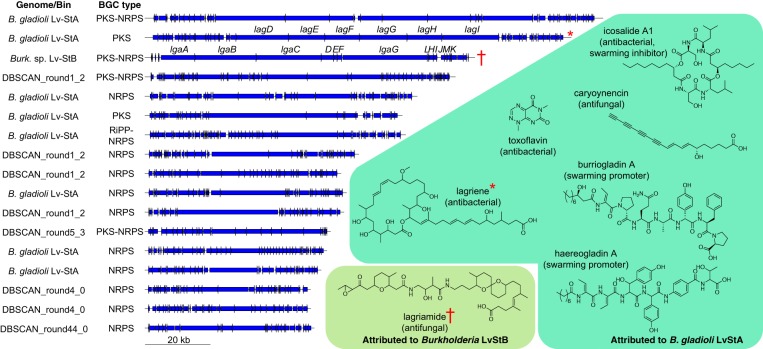
Schematics of all BGCs with length greater than 50 kbp assembled from the L. villosa metagenome and in the B. gladioli Lv-StA isolate genome among 126 identified by antiSMASH (left). Shown on the right are structures of compounds putatively assigned to BGCs in the B. gladioli Lv-StA genome (24, 25) or putatively assigned to a BGC in the *Burkholderia* sp. Lv-StB genome (6). Structures highlighted with red symbols have been attributed to the indicated BGCs.

Because we had previously isolated the nonreduced B. gladioli Lv-StA strain from L. villosa (6) and had found it to produce protective compounds despite having sporadic distribution and low abundance in field-collected beetles, we asked whether BGCs were a unique feature of Lv-StB in the metagenome or whether other community members have the biosynthetic machinery for potential chemical defenses. AntiSMASH (34) searches revealed a total of 105 BGCs in this metagenome, as well as 21 BGCs in the B. gladioli Lv-StA genome (Fig. 2), with variable BGC content ranging from 0 to 566 kbp per bin (0 to 14 BGCs, with 16 BGCs in the unclustered bin), while the B. gladioli Lv-StA genome contained 1,006-kbp BGCs. This indicates that Lv-StB is not the only strain in the egg microbiome with the potential to produce complex natural products and that many strains harbor multiple BGCs. In the metagenome, bins in the family *Burkholderiaceae* collectively contained the most BGCs by length (963 kbp), followed by a single bin in family *Rhodanobacteraceae* (DBSCAN_round1_2, 566 kbp, Fig. 2). This distribution suggests that although *Burkholderiaceae* appear to be an important reservoir of BGCs in the L. villosa egg microbiome, other groups have significant biosynthetic potential. Among the 126 BGCs detected in the metagenome and in the B. gladioli Lv-StA genome, there were 17 that were >50 kbp in length and were predicted to produce complex nonribosomal peptides or polyketides (Fig. 3). Two of these have been putatively assigned to production of the antibiotic lagriene in Lv-StA (24) and of the antifungal lagriamide in Lv-StB (6), whereas five other small molecules known to be produced by Lv-StA have been assigned to shorter BGCs (24, 25). We compared the 126 identified BGCs using BIG-SCAPE (35) and found only 7 examples of BGCs occurring in multiple strains, indicating that the biosynthetic potential in the metagenome and B. gladioli Lv-StA is largely nonredundant. Taken together, the data suggest that there is a large amount of undefined biosynthetic potential for production of potentially defensive small molecules in L. villosa symbionts, beyond B. gladioli Lv-StA and *Burkholderia* sp. Lv-StB.

### Divergence between Lv-StB and the closest free-living relative.

**FIG 4:**
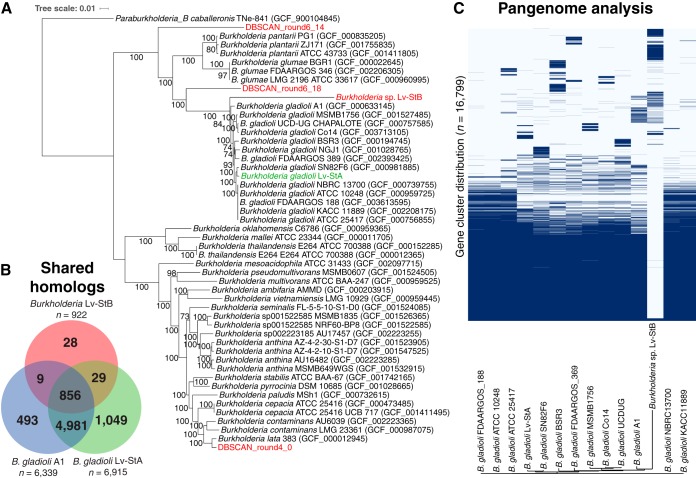
(A) Maximum-likelihood multilocus species tree of metagenomic bins classified in the genus *Burkholderia*, plus B. gladioli Lv-StB, using 120 marker gene protein sequences. Bootstrap proportions greater than 70% are indicated to the left of each node as a percentage of 1,000 replicates. Metagenomic bins are shown in red, while the L. villosa-associated isolate B. gladioli Lv-StA is shown in green. (B) Shared homologous gene groups in *Burkholderia* Lv-StA, B. gladioli A1, and B. gladioli Lv-StB, after discounting pseudogenes. Note that homologous groups are counted only once per genome (i.e., corresponding to collapsing paralogs), and therefore the indicated counts are lower than the absolute gene counts. Also, 492 genes in the Lv-StB genome were singletons and not included in homologous groups. (C) Hierarchical clustering of homologous gene clusters, showing presence and absence in *Burkholderia* sp. Lv-StB and closely related strains of B. gladioli.

We constructed a phylogenetic tree of metagenomic bins assigned to the genus *Burkholderia* as well as B. gladioli Lv-StA, on the basis of 120 marker genes (Fig. 4A). This showed that *Burkholderia* sp. Lv-StB is most highly related to the B. gladioli clade but is divergent from it. We calculated genome-wide ANI values for pairs of *Burkholderia* genomes and found that B. gladioli strains shared between 97% and 100% ANI whereas *Burkholderia* sp. Lv-StB shared at most 85.79% ANI with B. gladioli A1 (Data Set S1F). During genome reduction, symbionts are known to undergo rapid evolution due to the loss of DNA repair pathways (as found in the Lv-StB genome; see below) and the relaxation of selection (9), and so the divergence of Lv-StB from B. gladioli may have been accelerated relative to free-living lineages. Genome-reduced symbionts have often been vertically transmitted for evolutionary timescales and across host speciation events, and therefore it is possible to calculate evolution rates where related symbionts occur in hosts with known divergence times inferred from fossil records. Such estimates in insect symbionts range widely over 3 orders of magnitude, but more recent ant and sharp-shooter symbiont lineages (established <50 Mya for “*Candidatus* Baumannia cicadellinicola,” Blochmannia obliquus, Bl. pennsylvanicus, and Bl. floridanus) show high rates of divergence per synonymous site per year (dS/t) of between 1.1 × 10^−8^ and 8.9 × 10^−8^ (36) (the divergence rates used here can be found in Table S1 in the supplemental material). Because of the large number of pseudogenes in the Lv-StB genome, we reasoned that it is likely to be a recent symbiont and therefore used these rates to estimate times of divergence between Lv-StB and B. gladioli A1. We found a dS rate of 0.5486 per site between these genomes and calculated divergence times of 6.15, 8.55, 6.93, and 49.76 My rates for Bl. floridanus, Bl. pennsylvanicus, Bl. obliquus, and “*Candidatus* Baumannia cicadellinicola” divergence rates, respectively (Table S1) (36). We should note here that these time estimates are very approximate and are possibly overestimates as symbiont evolution rates are likely not constant, with particularly rapid evolution occurring during lifestyle transitions (11). The range of these estimated divergence times suggests that the common ancestor of *Burkholderia* sp. Lv-StB and B. gladioli existed after the evolution of symbiont-bearing structures in Lagriinae beetles (see below).

10.1128/mBio.02430-19.4 TABLE S1 Divergence rates used in this study (taken from a previous report by Silva and Santos-Garcia [36]). Download Table S1, DOCX file, 0.01 MB. Copyright © 2020 Waterworth et al. 2020 Waterworth et al. This content is distributed under the terms of the Creative Commons Attribution 4.0 International license.

We then sought to quantify the conservation of genes in Lv-StB compared to 13 related B. gladioli strains by identifying homologous gene groups in the entire set of nonpseudogenes in these strains with OMA (37). This pipeline aims to identify orthologous groups (OGs) while discounting paralogs among the genes in a given set of genomes (38). Of the 1,388 genes in Lv-StB that are not pseudogenes (Data Set S1B), 497 were not included in any OMA orthologous group (see below). A crude analysis of the OMA groups in Lv-StB, B. gladioli Lv-StA, and B. gladioli A1 revealed that Lv-StB retains a small subset of the groups found in both Lv-StA and A1 and has few unique groups (Fig. 4B), suggesting that Lv-StB has lost many of the genes conserved in B. gladioli. Consistent with this notion, we visualized the pangenome of B. gladioli and Lv-StB with Roary (39) (Fig. 4C) and found a large number of gene clusters that are conserved in B. gladioli but not Lv-StB. The gene clusters that are more variable among B. gladioli are also generally not found in Lv-StB. Conversely, there were gene clusters found in Lv-StB that are not present in B. gladioli strains, and these clusters may have been obtained by horizontal transfer after the divergence of Lv-StB or, alternatively, were lost in B. gladioli.

Remarkably, among the 1,149 pseudogenes detected in the Lv-StB genome, 976 were hypothetical and 129 were transposases, leaving only 44 that were recognizable (Data Set S1G). Pseudogenes were detected by identifying those that appeared truncated more than 20% compared to the closest BLAST hit (see Materials and Methods). While this methodology does not attempt to compare lengths to a consensus gene length (for example, to account for start site annotation variance) and while a firm cutoff for pseudogene length truncation has not been defined, the low number of recognizable pseudogenes suggests that we are not overannotating pseudogenes. The recognizable set of pseudogenes included a number of important genes in the categories noted to be depleted as described below. For instance, the DNA polymerase I (Pol I) gene (*polA*) appears to have been disrupted by a transposase, which is now flanked by two DNA Pol I fragments (E5299_1120 and E5299_01122). Likewise, the *uvrC* gene (E5299_00503), a component of the nucleotide excision repair system (40), is also present as a truncated gene adjacent to a transposase gene. There were also pseudogenes involved in the Entner-Doudoroff and glycolysis energy-producing pathways (corresponding to phosphogluconate dehydratase [41] and glucokinase [42]), as well as purine biosynthesis (corresponding to phosphoribosylglycinamide formyltransferase [43] and phosphoribosylformylglycinamidine synthase [44]). Interestingly, we found two pseudogenes that negatively regulate cell division and translation. Septum protein Maf is a nucleotide pyrophosphatase that has been shown to arrest cell division, especially after transformation or DNA damage (45). Deletion of the Escherichia coli gene for homolog YhdE increased the growth rate, while overexpression decreased the growth rate (46). Therefore, the loss of Maf in Lv-StB would be expected to increase the rate of cell division and reduce the conversion of nucleotides, which it probably obtains from the host, to the monophosphates. The gene for the energy-dependent translational throttle protein EttA was also found to be truncated. This protein slows translation through interacting with the ribosome in both the ATP- and ADP-bound forms (47, 48). Under energy-depleted conditions (i.e., high ADP levels), EttA was found to stabilize ribosomes and prevent commitment of metabolic resources; thus, the deletion mutant displayed reduced fitness during extended stationary phase (47). However, under circumstances where the host supplies ample nucleotides, the loss of *ettA* would be expected to increase translation rates.

### Degradation of primary metabolic pathways in *Burkholderia* sp. Lv-StB.

**FIG 5:**
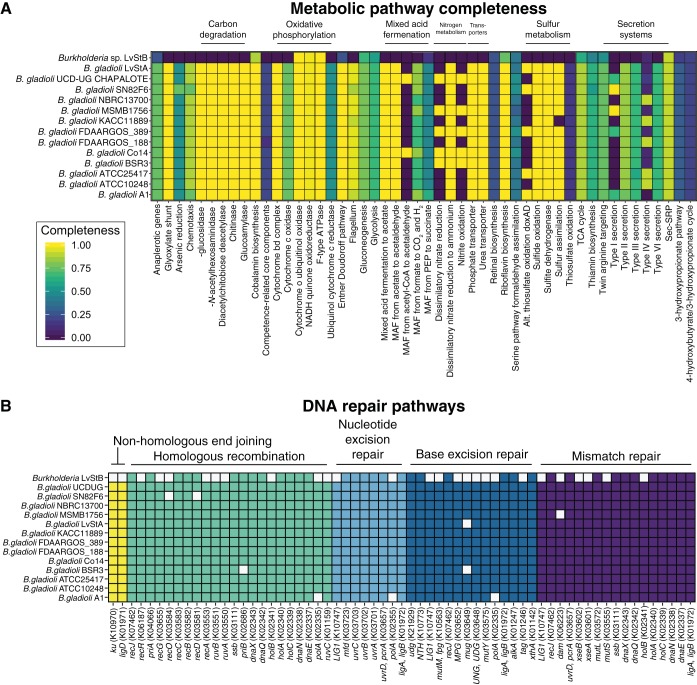
Completeness of metabolic and DNA repair pathways in *Burkholderia* sp. Lv-StB in comparison to closely related strains of B. gladioli. Note that presence/absence data shown here reflect detection in the draft-quality LvStB genome, and there is a possibility that genes are missing due to poor assembly. (A) Completeness of various metabolic pathways as determined by KEGG decoder. Note that categories that were not found in any of the examined genomes have been removed. MAF, mixed-acid fermentation; CoA, coenzyme A; TCA, tricarboxylic acid. (B) Presence (colored squares) and absence (white squares) of genes in the different DNA repair pathways in Lv-StB and related B. gladioli strains.

While the Lv-StB genome is of draft quality and thus may be missing some genes due to poor assembly, we found pervasive metabolic gaps across functional categories (Data Set S1A). Lv-StB is deficient in many metabolic pathways that are complete in related B. gladioli strains (Fig. 5), including the glyoxylate shunt (49) and various carbon degradation pathways as well as sulfur and nitrogen metabolism. Lv-StB appears incapable of making any of the following compounds due to the absence of several biosynthesis genes: thiamine, riboflavin, nicotinate, pantothenate, vitamin B12, and biotin. Likewise, there were deficiencies in amino acid biosynthesis (see Fig. S1 in the supplemental material). We predict that Lv-StB would be able to make chorismate, isoleucine, leucine, ornithine, proline, and threonine but likely lacks the ability to make aromatic amino acids, serine, methionine, lysine, histidine, cysteine, glutamine, and arginine due to the absence of several key genes in these pathways. The genome of Lv-StB also lacks genes involved in chemotaxis and flagella, suggesting that after the symbiont mixture is spread on eggs, the colonization of the dorsal cuticular structures in the embryo (24) does not require symbiont motility. Interestingly, the Lv-StB genome includes a trimeric autotransporter adhesin (TAA) related to SadA (50), which is involved in the pathogenicity of Salmonella enterica serovar Typhimurium by aiding cell aggregation, biofilm formation, and adhesion to human intestinal epithelial cells. TAAs are found in *Proteobacteria* and consist of anchor, stalk, and head domains, among which the head forms the adhesive component (51). The bacterial honey bee symbiont Snodgrassella alvi is hypothesized to utilize TAAs in combination with other extracellular components during colonization of the host gut (52), and similar genes were identified in S. alvi symbionts in bumble bees and are predicted to perform a similar role (53). Therefore, this gene may play a role in the adhesion of Lv-StB cells to L. villosa eggs.

10.1128/mBio.02430-19.1 FIG S1 Completeness of amino acid biosynthesis pathways in *Burkholderia* sp. Lv-StB in comparison to closely related strains of B. gladioli. Download FIG S1, EPS file, 1.8 MB. Copyright © 2020 Waterworth et al. 2020 Waterworth et al. This content is distributed under the terms of the Creative Commons Attribution 4.0 International license.

The Lv-StB genome is also missing several enzymes involved in glycolysis, most notably phosphoglycerate kinase and phosphoenolpyruvate carboxykinase (among others), which would suggest that Lv-StB has lost the ability to perform glycolysis. The loss of glycolysis is often compensated for by an alternative pathway, such as the pentose phosphate or Entner-Doudoroff pathway (54). This is not the case for Lv-StB, where both the glucose-6-phosphate dehydrogenase and 6-phosphogluconolactonase genes appear to be missing from the genome. The citrate cycle is largely complete, except that it is missing pyruvate carboxylase, the enzyme that converts pyruvate to oxaloacetate. However, phosphoenolpyruvate carboxylase is present in Lv-StB (E5299_00983) and may alternatively be used for the production of oxaloacetate from exogenous phosphoenolpyruvate (55), as alternative pathways for the supply of oxaloacetate are also incomplete. Lv-StB is also missing all genes required to form cytochrome *c* oxidase and cytochrome *b*-*c* complexes. However, all genes required to encode NADH:quinone oxidoreductase (complex I), succinate dehydrogenase (complex II), and cytochrome *o* ubiquinol oxidase are present, along with all genes required for the F-type ATPase. The lack of complex III would likely result in a decreased rate of ATP production in Lv-StB as observed in fungi with alternative oxidases that bypass complexes III and IV (56). Lv-StB may be similar to the psyllid endosymbiont “*Candidatus* Liberibacter asiaticus,” which has lost both key glycolysis genes and key glyoxalase genes and instead relies on the scavenging of ATP from the host (57).

We also found many deficiencies in both the *de novo* and salvage nucleotide pathways (Fig. S2). In the pyrimidine biosynthetic pathway, genes for dihydrooratase (*pyrC*) (58) and orotate phosphoribosyl transferase (*pyrE*) (59) were missing, suggesting that Lv-StB cannot produce orotate from *N-*carbamoylaspartate and cannot create nucleotides from free pyrimidine bases. Ribonucleotide reductase (*nrdAB*) (60) and thymidylate synthase (*thyA*) (61) are present, suggesting that deoxypyrimidine nucleotides can be made from CTP. The deficiencies in purine synthesis were more profound. The majority of the genes involved in the *de novo* pathway (62) were missing (*purCDEFHLMNT*, IMP dehydrogenase/*guaB*), except for those encoding adenylosuccinate lyase (*purB*), adenylosuccinate synthase (*purA*), and GMP synthase (*guaA*). Lv-StB should therefore be able to make AMP from IMP and GMP from XMP (plus their deoxy analogs through ribonucleotide reductase) but cannot make purines *de novo*. We were also not able to find adenine phosphoribosyltransferase or hypoxanthine-guanine phosphoribosyltransferase (62), meaning that purine bases cannot be salvaged to make nucleotides.

10.1128/mBio.02430-19.2 FIG S2 Completeness of nucleotide biosynthesis pathways in *Burkholderia* sp. Lv-StB in comparison to closely related strains of B. gladioli. Download FIG S2, EPS file, 2.5 MB. Copyright © 2020 Waterworth et al. 2020 Waterworth et al. This content is distributed under the terms of the Creative Commons Attribution 4.0 International license.

The loss of so many seemingly vital pathways would suggest that several key nutrients are acquired from either the host or other microbes. There are a total of 56 intact genes associated with transport in the Lv-StB genome (Table S2), of which 12 are annotated as efflux/export proteins. A total of 32 genes were found to be associated with ABC-type transporters and are predicted to be involved in the transport of carbohydrates, lipids, lipoproteins, cations, sugars, and methionine. Other genes were also found that encoded transporters that were predicted to transport biopolymers, potassium, osmoprotectants, and fructose. Finally, only broad identification could be achieved for the remaining transporters, including those within the major facilitator superfamily (MFS), the resistance-nodulation-cell division superfamily (RND), the drug/metabolite transporter superfamily, and the HlyC/CorC family transporter. It is therefore possible that many nutrients that cannot be created *de novo* within Lv-StB, such as the amino acid methionine, may be acquired from external sources, such as is observed between *Buchnera* bacteria and their aphid hosts (63).

10.1128/mBio.02430-19.5 TABLE S2 Intact transporter genes identified in the *Burkholderia* sp. Lv-StB genome. Download Table S2, DOCX file, 0.01 MB. Copyright © 2020 Waterworth et al. 2020 Waterworth et al. This content is distributed under the terms of the Creative Commons Attribution 4.0 International license.

### Degradation of DNA repair pathways in *Burkholderia* sp. Lv-StB.

The genome of Lv-StB is missing many genes involved in DNA repair (Fig. 5B), similarly to other examples of genome-reduced symbionts (9). Compared to closely related B. gladioli strains, Lv-StB lacks genes in every repair pathway. In particular, the DNA polymerase I gene (*polA*), involved in homologous recombination, nucleotide excision repair, and base excision repair, is present only as two truncated pseudogenes (see above). Even though *polA* is involved in many different DNA repair pathways, it has been found to be nonessential in E. coli (64, 65), B. pseudomallei (66), and B. cenocepacia (67). In the homologous recombination pathway, Lv-StB lacks *recA*, *polA*, *ruvA*, *ruvB*, *ruvC*, and *recG*, all of which have been found to be essential for homologous recombination in E. coli (68). Likewise, Lv-StB is also missing *ku* and *ligD*, the two components of the nonhomologous end-joining pathway (69), suggesting that it cannot recover from double-strand breaks. Lv-StB lacks several DNA glycosylases in the base excision repair pathway which are responsible for removing chemically modified bases from double-stranded DNA (70). Some of these losses in Lv-StB simply reduce redundancy, but it has also lost the nonredundant glycosylase genes *mutM* and *mug*. The former recognizes 2,6-diamino-4-hydroxy-5-*N*-methylformamidopyrimidine (Fapy) and 8-hydroxyguanine (71), while the latter recognizes G:U and G:T mismatches (72) as well as epsilonC (73), 8-HM-epsilonC (74), 1,N(2)-epsilonG (75), and 5-formyluracil (76). Finally, in the mismatch repair system, Lv-StB is missing *mutS*, which is required for the recognition of mismatches in methyl-directed repair (77). In summary, Lv-StB is likely to be completely incapable of nonhomologous end-joining, homologous recombination, and mismatch repair, while being impaired in nucleotide excision repair and base excision repair due to the loss of DNA polymerase I and several DNA glycosylases.

### Timing of horizontal acquisition of defensive and other genes in the Lv-StB genome.

**FIG 6:**
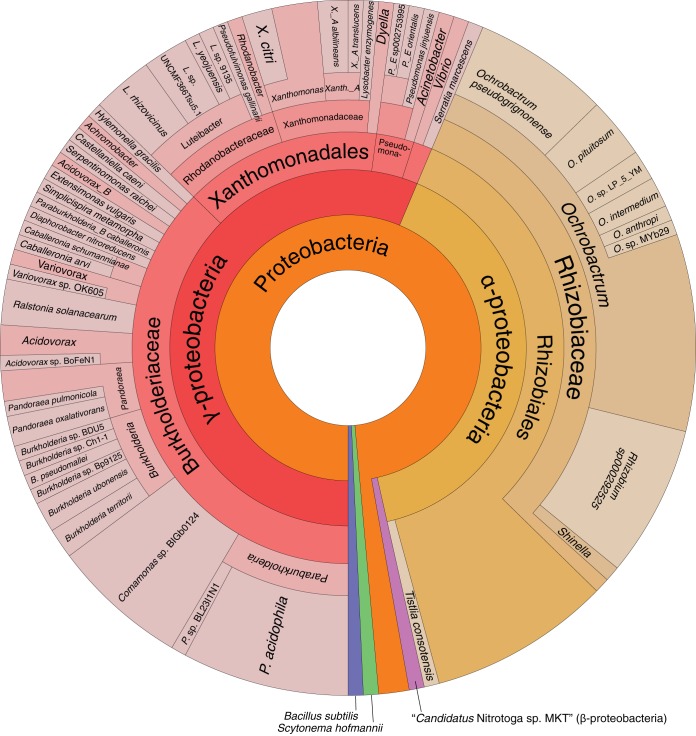
Putative sources of genes unique to the *Burkholderia* sp. Lv-StB genome (compared to B. gladioli strains) based on hits to BLASTP searches. Darker colors denote basal taxonomic classifications, while lighter colors denote specific taxonomic classifications. Note that three proteins of putative phage origin (see Data Set S1H) are not included in the figure.

We then attempted to identify genes in the *Burkholderia* sp. Lv-StB genome that are likely to have been acquired by horizontal gene transfer (HGT). A total of 497 intact genes were identified as unique to Lv-StB among 13 reference B. gladioli genomes (see Materials and Methods). Of these 497 genes, genes with no homologs in the NR database or genes that had homologs in B. gladioli genomes not included in this study were removed. A total of 148 genes appeared to be more closely related to species other than B. gladioli and may have been acquired through horizontal transfer (Fig. 6). A total of 79 and 57 genes appear to have been obtained from gammaproteobacteria and alphaproteobacteria, respectively, with one gene from a firmicute, one gene from a cyanobacterium, and three genes from phages (see Data Set S1G). The distribution is consistent with the notion that horizontal transfer occurs most frequently between closely related species (78). In particular, *Burkholderiaceae* species were the most frequent apparent donors of gammaproteobacterial proteins. Interestingly, the alphaproteobacterial genus *Ochrobactrum* (family *Brucellaceae* in the NCBI taxonomy and *Rhizobiaceae* in GTDB) was a major putative gene donor. This genus includes several symbionts of termites (79), army worms (80), weevils (81), and leeches (82, 83). In previous 16S amplicon investigations of *Lagria* beetles, *Ochrobactrum* spp. were often found (24, 26) (Fig. S3), suggesting that the donors of these genes could have also been associates of L. villosa. *Ochrobactrum* operational taxonomic units (OTUs) account for 5% to 20% of 16S rRNA gene reads, but this genus was not observed in the shotgun metagenome. However, disparities between 16S and shotgun metagenome abundances are not uncommon due to variable 16S copy numbers and primer and sequencing biases (84, 85). On the basis of the evidence for putatively horizontally transferred genes, we asked whether these could have contributed to the dominance of Lv-StB in L. villosa and set out to estimate the timings of horizontal transfer events.

10.1128/mBio.02430-19.3 FIG S3 Reanalysis of 16S rRNA amplicon data reported previously by Flórez et al. (24), showing the distribution of dominant microbial communities associated with L. villosa egg clusters. Amplicon 16S rRNA gene sequences were clustered into operational taxonomic units (OTUs) at a distance of 0.03, an approximation used for bacterial species. The putative taxonomic classification of each OTU is indicated with a colored key. The relative proportions of the top 10 most abundant OTUs are indicated as percentages of the total reads for each cluster of eggs collected. Download FIG S3, EPS file, 1.6 MB. Copyright © 2020 Waterworth et al. 2020 Waterworth et al. This content is distributed under the terms of the Creative Commons Attribution 4.0 International license.

Horizontally transferred genes are often detected on the basis of nucleotide composition differing from that of other genes in the genome (86). Such genes initially exhibit nucleotide composition consistent with the donor genome, which eventually normalizes to the composition of the recipient genome (87). The rate of this “amelioration” process (ΔGC^HT^) has been modeled by Lawrence and Ochman (87), based on the substitution rate (*S*), the transition/transversion ratio (κ), and the GC content of both the recipient genome (GC^EQ^) and the putatively horizontally transferred genes (GC^HT^), according to equation 1. By iterating this equation repeatedly until GC^HT^ equals GC^EQ^, the time required from the present day to complete amelioration can be estimated. If the GC content of the donor genome is known, then equation 1 can be used in reverse to estimate the time since introgression. However, if the donor GC content is not known, then the differing selection pressures on the first, second, and third codon positions can be exploited to estimate the introgression time. Because these positions have different degrees of amino acid degeneracy, they are subject to different degrees of selection, and therefore they ameliorate at different rates. As a consequence, Lawrence and Ochman (87) found that for genes in the process of amelioration, the relationship between overall GC content and the GC content at individual codon positions seen in genes at equilibrium (87, 88) (equations 2, 3, and 4) does not hold. So if equation 1 is applied in reverse separately for each codon position, the time since introgression can be inferred at the iteration yielding the minimum square difference from equations 2 to 4. Application of equation 1 also yields an estimate for the original donor GC.

(1) $$\Delta {\text{GC}}^{\text{HT}}=S\times \frac{\kappa +\frac{1}{2}}{\kappa +1}\times \left[{\text{GC}}^{\text{EQ}}-{\text{GC}}^{\text{HT}}\right]$$

(2) $${\text{GC}}_{\text{1st}}=0.615\times {\text{GC}}_{\text{genome}}+26.9$$

(3) $${\text{GC}}_{\text{2nd}}=0.270\times {\text{GC}}_{\text{genome}}+26.7$$

(4) $${\text{GC}}_{\text{3rd}}=1.692\times {\text{GC}}_{\text{genome}}-32.3$$

**FIG 7:**
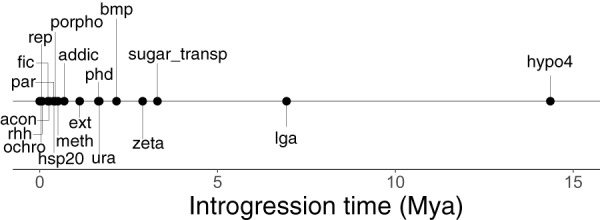
Estimated introgression time for putative HGT gene sets, using the “*Candidatus* Baumannia cicadellinicola” substitution rates (36). Note that for clarity, the gene sets with estimated introgression time of <5,000 ya are not labeled. For these data and ages estimated with other substitution rates, see Data Set S1H. Abbreviations refer to groups of genes as outlined in Data Set S1H: acon, E5299_02570–E5299_2571; addic, E5299_02248–E5299_02250; meth, E5299_02474–E5299_02475; ochro, E5299_01926–E5299_01933; par, E5299_02349–E5299_02352; phd, E5299_02420–E5299_02423; porfo, E5299_02300, E5299_02302–E5299_02303; rep, E5299_02219–E5299_02220; rhh, E5299_01720, E5299_01726; ura, E5299_02533–E5299_02435.

We identified groups of consecutive genes in the putative HGT set that could have been acquired together and used the method described above to estimate their introgression time (Data Set S1H). Among the 18 identified gene groups, 7 were found to have atypical GC content (defined by Lawrence and Ochman [87]), with GC% content that was either >10% lower or >8% higher at the first codon position or the third codon position, respectively, than that of the genome as a whole. The method described above was used to estimate the time of introgression for each gene group, using the Bl. floridanus, Bl. pennsylvanicus, Bl. obliquus, and “*Ca.* Baumannia cicadellinicola” divergence rates (see above and Fig. 7; see also Data Set S1H). The oldest horizontal transfer (1.79 to 14.36 Mya) was found to correspond to the “hypo4” group (E5299_02488–E5299_02489), representing two phage proteins, and the next oldest corresponded to the lagriamide BGC and neighboring genes (*lga*; 0.8 to 6.42 Mya; E5299_00001–E5299_00003, E5299_00005–E5299_00018). The *lga* BGC is predicted to have come from a high-GC organism, with original GC content of 72%. The closest relatives of many of the *lga* genes are found in *Pseudomonas* strains, which typically do not have GC content this high. However, as BGCs are often thought to be horizontally transferred (89), *Pseudomonas* may not be the direct source of *lga* in Lv-StB.

Several other HGT gene groups (sugar_transp [E5299_01760–E5299_01761, E5299_01763], bmp [E5299_02444], ext [E5299_02524–E5299_02526], dinj [E5299_02222, E5299_02224], tonb [E5299_00736–E5299_00737], tonb2 [E5299_02080]) were predicted to be involved in transport, with predicted substrates that included purine nucleosides, trehalose, and vitamin B12. Of these, the sugar_transp and bmp groups, predicted to be involved in importation of trehalose and purine, respectively, are relatively old (0.41 to 3.31 My and 0.27–2.16 My, respectively), and the groups likely involved in B12 importation (dinj, tonb, tonb2) are estimated to be <5,000 y old. Trehalose is the most abundant component of insect hemolymph, and was found to be provided by the host to its genome-reduced symbiont in a leaf beetle system (90). We also found HGT gene groups putatively involved in heat shock response (hsp20; 0.05 to 0.4 Mya) and DNA repair (ura [E5299_02533–E5299_02535], rep [E5299_02219–E5299_02220], ochro [E5299_01926–E5299_01933]). In the latter category, we identified a uracil-DNA glycosylase (in ura; 0.21 to 1.68 Mya), used in base excision repair in cases of deamination; RecB, used in homologous recombination (in rep; 0.01 to 0.055 Mya) (62); and YedK (in ochro; 0.01 to 0.035 Mya) (91), a protein used in the SOS response that binds to abasic sites in single-stranded DNA. The transferred transport functions and DNA repair proteins match functional categories that are currently lacking genes due to genome reduction (see above), and therefore the transfers could have been contemporaneous with the reduction process, acting as mechanisms compensating for lost functions.

One putative HGT toxin that is unique to Lv-StB in the metagenome and among B. gladioli strains is zonular occludens toxin (zot [E5299_02544–E5299_02545]; estimated to have been acquired <5,000 ya). The *zot* gene is responsible for the production of the zonula occludens toxin, a virulence factor which was initially identified in Vibrio cholerae and whose presence was found to lead to the disassembly of intracellular tight junctions and consequently to increased permeability of mammalian epithelium (92). Colocalized with the predicted *zot* gene was a gene encoding DUF2523, which we found often accompanied *zot* in our searches of the STRING database (93). Zot proteins have been identified in several strains of *Campylobacter* and have been shown to elicit an inflammatory response in intestinal epithelial cells (94, 95). Furthermore, a significant correlation was found between the presence of the Zot protein and hyperinvasive strains of Neisseria meningitidis (96). Potentially, *zot* may aid in the infection of the L. villosa embryonic structures through increasing permeability across the outer layers of the egg, although this remains speculative.

### The evolution of *Burkholderia* sp. Lv-StB.

In the 1920s it was observed (97) that beetles in the *Lagria* and *Cerogria* genera possessed structures now known to harbor *Burkholderia* symbionts in L. villosa and *L. hirta* (26). Other genera in the Lagriinae subfamily, such as *Adynata* and *Arthromacra*, do not have these structures. According to the tree found at timetree.org (98), *Lagria* and *Cerogria* diverged 55 Mya, and the common ancestor of *Lagria*, *Cerogria*, and *Adynata* existed 82 Mya (this region of the tree utilizes data from Kergoat et al. [99]). On the basis of these estimates, the symbiont-bearing structures in *Lagria* and *Cerogria* likely evolved between 82 and 55 Mya. Our analysis suggests that the divergence of *Burkholderia* sp. Lv-StB from B. gladioli occurred after that point (6.15 to 49.76 Mya). During that time, the genome of *Burkholderia* sp. Lv-StB became reduced, and it is likely dependent on the host due to deficiencies in energy metabolism and nucleotide biosynthesis. Notably, the profound metabolic insufficiencies and incomplete DNA repair pathways in Lv-StB are typical of symbionts with smaller genomes, such as “*Ca.* Endolissoclinum faulkneri,” an intracellular tunicate symbiont with a 1.48-Mbp genome and a similar number of genes (783) (100), estimated to have been a symbiont for at least 6 to 31 My (101). While the presence of *Burkholderia* sp. Lv-StB and of its defensive compound lagriamide has been shown to decrease the rate of fungal egg infection (6), the symbiont is not essential for beetle reproduction (24). Therefore, the relationship is facultative from the perspective of the host, while Lv-StB is in the process of becoming dependent on L. villosa. A central issue that we aimed to address in this work was that of how the genome of Lv-StB became reduced, when L. villosa appears to maintain multiple other nonreduced *Burkholderia* spp. and other symbionts.

It is clear from previous work that the *Lagria* symbionts related to B. gladioli evolved from plant-associated strains (26), likely transmitted to the insects from the plant environment. Intriguingly, there are closely related *Burkholderia* (now formally *Caballeronia*) symbionts of several plant species within the Rubiaceae and Primulaceae families which reside within the host’s leaf nodules (102) and have genomes that are similar in size to that of *Burkholderia* sp. Lv-StB. In “*Ca.* Burkholderia crenata,” hosted by the plant *Ardisia crenata*, there are a number of interesting parallels with *Burkholderia* sp. Lv-StB, such as the maintenance of secondary metabolite BGCs and loss of essential genes and DNA repair functions (103). Interestingly, lateral gene transfers of the secondary metabolite BGCs between different symbionts have occurred, likely after transition to a host-restricted lifestyle (104). It is possible that the evolution of the L. villosa symbiont resembles that of the leaf nodulating *Burkholderia* symbionts given the similarities in function, genome characteristics, and degrees of host dependence, although “*Ca.* Burkholderia crenata” has the smallest genome of leaf nodule symbionts and, in contrast to Lv-StB, maintains the capability of synthesizing most cofactors and vitamins.

For the *Lagria* host, the probable advantage of the consistent presence of *Burkholderia* or other bacteria is that of protection of eggs from infection, through the activity of small molecules made by its microbiome. The strains characterized here, as well as the previously isolated Lv-StA strain, were found to contain ample biosynthetic potential, and both Lv-StA and Lv-StB produce antifungal compounds that were found to protect eggs from fungal infection in laboratory experiments (26). And yet, Lv-StA is found only sporadically as a minor component of the microbiome in the field (6). It is probably advantageous for *Lagria* beetles to maintain a pool of facultative symbionts with different biosynthetic capabilities to allow fast adaptation to different environmental infection pressures (105). However, there may be less selection pressure on a facultative symbiont to stay associated with its host if it can also survive in the environment and infect plants.

The foundational event in the establishment of the symbiosis between Lv-StB and L. villosa was likely the acquisition of the *lga* pathway, which putatively produces lagriamide, in a nonreduced ancestor of Lv-StB (6). We place this as the first event for four reasons. First, the *lga* BGC is almost the oldest detectable horizontal transfer that survives in the reduced genome of Lv-StB. Second, we found little evidence that Lv-StB is capable of making metabolites of use to the host, indicating that the symbiosis is likely not based on nutrition. L. villosa’s diet of plant leaves may be nitrogen poor, with hard-to-digest plant cell wall components (106), but we did not find polysaccharide-degrading pathways or pathways corresponding to extensive biosynthesis of essential amino acids in the Lv-StB genome. Therefore, the *lga* BGC is the oldest remaining feature that potentially increases host fitness. Third, the reduced coding density seen in the Lv-StB genome may be indicative of a recent transitional event (11), such as strict host association or a move to vertical transmission. Fourth, even though we found genes missing from all DNA repair pathways, which is thought to be a driver for increased AT content in symbiont genomes (9), and increases in AT content may have an adaptive component that reduces the metabolic costs associated with symbionts (23), the GC content of the Lv-StB genome is not very different from that of free-living B. gladioli strains in comparison to other “transitional” symbionts. For instance, “*Candidatus* Pantoea carbekii” has a reduced genome and, like Lv-StB, lacks full-length DNA polymerase I (107). The GC content of this strain is almost 30% lower than that of its closest free-living relatives (11), while the GC content of the genome of Lv-StB is ∼10% lower than that of its closest relatives. Therefore, we propose that loss of DNA repair pathways and other genome degradation events in Lv-StB occurred relatively recently, after the acquisition of *lga*.

It can be envisioned that *lga* provided a sustained survival advantage in an environment where lagriamide reduced egg fungal infections and that there was positive selection in beetles that vertically transmitted *lga*-bearing symbionts. It is also possible, although speculative, that production of lagriamide confers a competitive advantage among the members of the microbial community in the beetle. In L. villosa, symbionts are stored extracellularly, and they are spread onto the exterior of eggs as they are laid. According to observations in the congeneric species *L. hirta*, the symbionts are assumed to first enter through the egg micropyle to reach the embryonic organs where they are housed throughout larval development (97). It is thus likely that only a few of the cells are vertically transmitted as a result of colonizing these structures, potentially providing the population bottlenecks that could have caused initial accumulation of deleterious mutations that started the process of genome reduction. Meanwhile, loss of certain proteins limiting growth rate (see above) may have been selected through the presence of increased Lv-StB populations and compound production. It is unknown to what extent Lv-StB is genetically isolated in the larval or adult host, but we found evidence of ongoing horizontal transfer events having occurred in the recent past, presumably through contact with a complex microbiome associated with L. villosa egg surfaces. These horizontal gene transfers likely happened concurrently with the ongoing genome reduction process and may have been compensatory for gene losses. There is some precedence for the idea of the existence of extracellular symbionts with profoundly reduced genomes (106, 108–110). For instance, the leaf beetle *Cassida rubiginosa* harbors “*Candidatus* Stammera capleta,” a symbiont with the smallest genome of any extracellular organism (0.27 Mbp), which provides pectinolytic enzymes to help break down the host’s leafy diet (106). In many of these cases, symbionts are stored as isolated monocultures within specialized structures in adult hosts, while vertical transmission is assisted by packaging symbiont cells in protective “caplets” attached to eggs (106) or in a “symbiont capsule” encased in chitin (108) or by their secretion in a galactan-based jelly ingested by hatched juveniles (110), although reduced-genome symbionts have also been known to be vertically transmitted by simple egg surface contamination (109), as in L. villosa. Because these examples represent advanced cases of genome reduction, it would be difficult to determine whether horizontal transfer events occurred before or during genome reduction, and none have been noted. The complexity of the L. villosa microbiome appears to represent a different case, as it afforded ample opportunity for horizontal gene transfer even while the genome of Lv-StB was actively undergoing reduction.

In addition to the leaf nodule *Burkholderia* symbionts (see above), horizontal acquisition of genes has been observed in two types of reduced-genome symbionts, namely, eukaryotic parasites in the genus *Encephalitozoon* (111) and *Acetobacteraceae* strains associated with the gut community of red carpenter ants (112). In both of these cases, symbiont genomes were similar in size to Lv-StB (∼2 Mbp) but showed far greater coding density and fewer pseudogenes. Furthermore, both the *Encephalitozoon* and *Acetobacteraceae* strains were culturable, suggesting that they are facultative symbionts in a less advanced state of genome reduction than Lv-StB. The genome of Lv-StB appears to be different from the genomes of these examples, because there is evidence of recent horizontal transfers, even though genes required for homologous recombination are currently missing. Either the loss of homologous recombination was very recent or such transfers could have occurred in a RecA-independent manner. For example, plasmids could have been transferred into Lv-StB cells, followed by the RecA-independent transposition of genes to the chromosome (113, 114). Another potential mechanism is phage infection. For instance, it is known that the genomes of symbionts that switch hosts such as *Wolbachia* strains tend to contain a large amount of mobile DNA and phage remnants (115).

### Conclusions.

It is unclear whether Lv-StB will continue on the path of genome reduction to become drastically reduced with a <1-Mbp genome. Where symbionts are required for host survival and are genetically isolated within host cells or specialized structures, such a process appears to be irreversible and unstoppable (116, 117). However, a number of alternate fates could be envisioned for Lv-StB. With a complex microbiome, if ongoing gene losses in Lv-StB reduce its fitness past a certain point, then it could be replaced by another strain, potentially accompanied by horizontal transfer of the *lga* pathway to a less reduced genomic chassis. Alternatively, horizontal transfers of genes to Lv-StB could lead to an equilibrium of gene loss and gain. Interestingly, it appears that until the present time, horizontal transfer had not occurred fast enough to prevent widespread loss of metabolism and DNA repair in the Lv-StB genome. The host could also evolve strategies to maintain increasingly genome-reduced Lv-StB, perhaps by selective extracellular partitioning and packaging for vertical transfer similarly to the examples outlined above. However, it is unclear whether such an evolutionary path would be favorable, given that the coinfection of multiple BGC-bearing symbiont strains could be advantageous in environments with variable pathogen pressures.

In summary, evidence gathered here suggests that the introduction of the lagriamide BGC preceded and perhaps initiated genome erosion of Lv-StB, potentially through selection of beetles that transferred the symbiont vertically, leading to a population structure with frequent bottlenecks. Simultaneous advantageous gene acquisitions may have enabled the preferential survival of Lv-StB and its dominance in the adult host and the egg surface.

## MATERIALS AND METHODS

### Sequencing and assembly of Burkholderia gladioli Lv-StA genome.

Genome sequencing of the isolated B. gladioli Lv-StA strain was carried out using PacBio with single-molecule, real-time (SMRT) technology. For *de novo* assembly (carried out by Eurofins Genomics), the HGAP pipeline was used (Heirarchical Genome Assembly Process). Briefly, a preassembly of long and accurate sequences was generated by mapping filtered subreads to so-called seed reads. Subsequently, the Celera assembler was used to generate a draft assembly using multikilobase long reads, which in this case rendered full-genome closure. Finally, the Quiver algorithm was used to correct indel and substitution errors by considering the quality values from the bas.h5 files.

### Metagenomic binning and annotation.

Metagenomic assembly files were clustered into putative genomic bins using Autometa (Master branch—commit bbcea30) (28). Contigs with lengths shorter than 3,000 bp were excluded from the binning process, and a taxonomy table was produced. Contigs classified as bacterial were further binned into putative genomic bins using run_autometa.py. Unclustered contigs were recruited into clusters using ML_recruitment.py. Results were summarized using cluster_process.py. The resultant genome bins were compared to earlier versions (6) using Mash version 2.1.1 (118), which hashes genomes to patterns of *k*-mers (sketching), allowing rapid calculations of distances between two sketches. All bins were sketched, and distances were computed in a pairwise fashion. Pairwise distances were visualized in R as a dendrogram and enabled the determination of equivalent old and updated putative genome bins between analyses. The updated putative genomic bins were annotated using Prokka version 1.13 (119), with GenBank compliance enabled. Reference genomes downloaded from NCBI were similarly annotated with Prokka in order to maintain consistency between data sets. Amino acid sequences of open-reading frames (ORFs) were further annotated using DIAMOND blastp version 0.9.21.122 (120) against the Diamond formatted NR database. The search was limited to returning a maximum of 1 target sequence, and the maximum number of high-scoring pairs per subject sequence was set to 1. Results were summarized in BLAST tabular format with qseqid (Query gene identifier [ID]), stitle (aligned gene ID), pident (percentage of identical matches), evalue (expected value), qlen (query sequence length), and slen (aligned gene sequence length) as desired parameter output. Pseudogenes were identified by finding Lv-StB genes that were more than 20% shorter than their respective BLAST matches. This criterion has been used previously to identify pseudogenes (101, 121).

Coding density was calculated as the sum of all protein-coding sequences (coding sequence) as a percentage of the sum of all contigs (total sequence). In cases where protein-coding genes were found to overlap, the length of the overlap region was counted only once. This calculation was performed for all binned genomes, both on the initial data sets (GenBank files generated during Prokka annotations) and on the edited data sets where pseudogenes had been removed. For the identification and count of genes encoding transposases and hypothetical proteins, protein-coding gene amino acid files (*.faa) containing Prokka annotations were parsed for gene descriptions containing “transposase” and “hypothetical” strings.

### Taxonomic classification of genome bins.

Putative genome bins clustered from the L. villosa metagenomic data set were taxonomically classified using GTDB-Tk v0.2.2 (reference database gtdbtk.r86_v2) with default parameters (29). GTDB-Tk identifies and aligns 120 bacterial marker genes per genome before calculating the optimal placement of the respective alignments in the precomputed GTDB-Tk reference tree which consists of 94,759 genomes (see Data Set S1B in the supplemental material). A species was assigned to a genome if it shared 95% or more ANI with a reference genome.

### Identification of “core” genes.

The set of “core” genes generally found in even the most reduced symbiont genomes was taken from the data provided in Table 2 of a previous report by McCutcheon and Moran (9). GFF files produced for B. gladioli Lv-StA and the metagenomic bins by Prokka were searched for the following gene designations: “*dnaE*,” “*dnaQ*,” “*rpoA*,” “*rpoB*,” “*rpoC*,” “*rpoD*,” “*groL*,” “*groS*,” “*dnaK*,” “*mnmA*,” “*mnmE*,” “*mnmG*,” “*sufS*,” “*sufB*,” “*sufC*,” “*iscS*,” “*iscA*,” “*iscU*,” “*rluA*,” “*rluB*,” “*rluC*,” “*rluD*,” “*rluE*,” “*rluF*,” “*infA*,” “*infB*,” “*infC*,” “*fusA*,” “*tsf*,” “*prfA*,” “*prfB*,” “*frr*,” “*def*,” “*alaS*,” “*gltX*,” “*glyQ*,” “*ileS*,” “*metG*,” “*pheS*,” “*trpS*,” “*valS*,” “*rpsA*,” “*rpsB*,” “*rpsC*,” “*rpsD*,” “*rpsE*,” “*rpsG*,” “*rpsH*,” “*rpsI*,” “*rpsJ*,” “*rpsK*,” “*rpsL*,” “*rpsM*,” “*rpsN*,” “*rpsP*,” “*rpsQ*,” “*rpsR*,” “*rpsS*,” “*rplB*,” “*rplC*,” “*rplD*,” “*rplE*,” “*rplF*,” “*rplK*,” “*rplM*,” “*rplN*,” “*rplO*,” “*rplP*,” “*rplT*,” “*rplV*,” “*rpmA*,” “*rpmB*,” “*rpmG*,” “*rpmJ*,” “*tRNA-Met*,” “*tRNA-Gly*,” “*tRNA-Cys*,” “*tRNA-Phe*,” “*tRNA-Lys*,” “*tRNA-Ala*,” “*tRNA-Glu*,” “*tRNA-Pro*,” “*tRNA-Gln*,” and “*tRNA-Ile*.” The presence of single or multiple examples of these genes per genome/bin was tabulated in Microsoft Excel to produce Data Set S1D, and the percentage of the core gene set found in a genome/bin was used for Table 1.

### Annotation and analysis of BGCs.

Putative biosynthetic gene clusters were identified in all binned genomes using the AntiSMASH (122) docker image (image ID: 8942d142d9ac). HMMer analysis of the entire genome was enabled, and identified clusters were compared to both antiSMASH-predicted clusters, the MIBiG database, and secondary-metabolite orthologous groups. Similarities between identified putative biosynthetic gene clusters were assessed using BiG-SCAPE version 20181005 (35) in “glocal” mode.

### Construction of multilocus species tree.

Genomes of *Burkholderia* sp. Lv-StB and B. gladioli Lv-StA and binned genomes taxonomically classified within the *Burkholderia* genus, including DBSCAN_round6_14, DBSCAN_round6_18, and DBSCAN_round4_0, were uploaded to the AutoMLST website (123). A concatenated species tree was constructed in *de novo* mode (with default options), with the IQ-TREE Ultrafast Bootstrap analysis and ModelFinder options enabled.

### Calculation of average nucleotide identities.

The average nucleotide identities (ANIs) of *Burkholderia* sp. Lv-StB and B. gladioli were calculated in a pairwise manner using FastANI (124) against 45 *Burkholderia* reference genomes downloaded from NCBI. A total of 13 genomes shared over 95% ANI (Data Set S1F). These genomes were all identified as B. gladioli species and were used in downstream analyses.

### Quantification of divergence between *Burkholderia* sp. Lv-StB and B. gladioli A1.

Orthologous protein sequences were identified in nonpseudogene sequence files of *Burkholderia* sp. Lv-StB and 13 closely related genomes (B. gladioli Lv-StA, B. gladioli A1, B. gladioli UCDUG, B. gladioli FDAARGOS_389, B. gladioli ATCC 25417, B. gladioli Co14, B. gladioli SN82F6, B. gladioli ATCC 10248, B. gladioli NBRC13700, B. gladioli FDAARGOS_188, B. gladioli MSMB1756, B. gladioli BSR3, and B. gladioli KACC11889) identified through the ANI analyses using OMA version 2.2.0 (37). A subset (797 groups) of the resultant orthologous groups (OGs) was identified which included genes from all 14 genomes used in the analysis. Each set of OG sequences were aligned using MUSCLE v3.8.31 (125), and the corresponding nucleotide files were extracted and aligned against the amino acid sequences using the PAL2NAL docker image (image ID: ce3b1d7d83ab) (126) using codon table 11 and specifying no gaps, with paml as the output format. The resultant paml files were used to estimate pairwise *dS* (synonymous divergence rate), *dN* (nonsynonymous divergence rate), and kappa (transition/transversion ratio) values from comparisons between individual genes per orthologous group with codeml (127) in the PAL2NAL package. The following parameters were specified in the control file: runmode = −2 (pairwise), model = 0 (one), fix_kappa = 0 (kappa to be estimated), and fix_omega = 0 (estimate omega, where omega is the *dN*/*dS* ratio, with initial omega set to 0.2). Any orthologous gene sets that included genes that gave a *dS* value over 3 were removed from the analysis (128). Individual sequences from remaining OGs were then gathered into genome-specific files (i.e., all Lv-StB genes in all OGs were moved into an ordered Lv-StB.faa/.ffn file). Stop codons were removed from each nucleotide sequence. Sequences were then concatenated per genome to produce a single sequence per genome. The concatenated amino acid sequences and corresponding nucleotide sequences were aligned against one another using PAL2NAL as performed for individual genes. Pairwise estimations of *dS*, *dN*, and kappa were calculated as before using codeml. Additionally, the concatenated genes were analyzed a second time using an alternative control file, in which the model was set to 2. The likelihood ratio test value for comparisons between pairwise null and alternative likelihood scores was calculated (2 × Alt_lnl − Null_lnl) for Lv-StB relative to the 13 reference genomes and found to be 0 in all cases, indicating that the omega (*dN*/*dS*) ratio was consistent in comparisons between Lv-StB and the reference genomes. Individual *dS* values were used to estimate divergence between Lv-StB and the 13 reference genomes with divergence rates estimated using the method described previously by Silva and Santos-Garcia (36) (see Table S1 in the supplemental material) in the following equation: age of divergence (Mya) = *dS* ÷ divergence rate × 1,000,000. As Lv-StB shared the greatest ANI with B. gladioli A1, the kappa value found in comparisons between these two genomes (7.03487) was used for amelioration estimates.

### Pangenome analysis.

To assess the pangenome of Lv-StB and other B. gladioli genomes, GFF files generated by Prokka were analyzed using Roary (39), which identifies core and accessory genes per genome. Concatenation and alignment of orthologous genes were enabled in Roary and used to build a phylogenetic tree with FastTree version 2.1.10 (129). The resultant phylogenetic tree and presence/absence matrix of genes in all genomes were visualized with the roary_plots.py script. Additionally, nonpseudogenes of all genomes were annotated against the KEGG database (130, 131) using kofamscan (132) with the output in mapper format. An overview of the completeness of general metabolic pathways was visualized using KEGG-Decoder (133) with kofamscan annotations. For specific pathways of interest (amino acids, DNA repair, nucleotide *de novo* biosynthesis), presence/absence matrices of genes per KEGG pathway entry were visualized in R version 3.6.0 using the tidyr, ggplot2, and viridis libraries.

### Identification of genes putatively acquired by horizontal transfer.

All amino acid sequence files representing nonpseudogenes of all genomes used in the ANI analysis (B. gladioli Lv-StA and 45 *Burkholderia* reference genomes) were concatenated and converted into a DIAMOND BLAST (120) database (build 125). The amino acid sequence files of nonpseudogenes in Lv-StB were then searched against this database. All genes that had no significant hit, or that had a significant hit but with a shared proportion of less than 50%, were considered unique to Lv-StB. Nonpseudogenes of Lv-StB that were not found to have an ortholog in the OMA analysis were used to validate this list. These unique genes were then compared to the NR database using DIAMOND blastp (as described above), and any genes that had no significant hit were removed from the set of unique genes. Manual inspection of the remaining genes resulted in the removal of any genes that were closely related to B. gladioli genes (i.e., were found within B. gladioli genomes other than the ones investigated here). The remaining unique genes were consequently considered to represent genes potentially acquired via horizontal transfer. This list was expanded with other genes within Lv-StB, for which homologs in *Burkholderia* genomes could be found, but the closest match against the NR database belonged to a different genus. For example, E5299_02249 of the “addic” group shared 53.1% sequence identity with a gene from *B. contaminans* strain LMG 23361 but shared a higher sequence identity level of 96.9% with Ochrobactrum pituitosum.

### Deamelioration of putatively horizontally transferred genes.

The method of Lawrence and Ochman (87) was implemented in Python and is available at https://bitbucket.org/jason_c_kwan/age_horizontal_transfers.py. The script takes as input (i) an in-frame nucleotide FASTA file containing the sequences of putatively horizontally transferred genes, (ii) an in-frame nucleotide FASTA file containing a comparison set of gene sequences from the genome, (iii) a synonymous mutation rate in substitutions per 100 sites per million years, (iv) a nonsynonymous mutation rate in substitutions per 100 sites per million years, (v) a transition/transversion ratio (κ), (vi) a step time in millions of years, and (vii) a maximum time to iterate to. The script outputs GC content of each codon position (plus overall GC) at each time point and reports the estimated age of the gene cluster corresponding to the iteration with the smallest sum of squared deviations from equations 2 to 4. The substitution rates used in our calculations were half of the divergence rates estimated by Silva and Santos-Garcia (36) and were as follows: for “*Ca.* Ba. cicadellinicola,” synonymous rate of 0.55 and nonsynonymous rate of 0.05; for Bl. obliquus, synonymous rate of 3.95 and nonsynonymous rate of 0.26; for Bl. pennsylvanicus, synonymous rate of 3.2 and nonsynonymous rate of 0.28; for Bl. floridanus, synonymous rate of 4.45 and nonsynonymous rate of 0.395. A value of 7.0348 was used for κ, previously calculated for *Burkholderia* sp. Lv-StB and B. gladioli A1 (see above). A step time of 0.005 My and a maximum time of 50 My were used in all calculations. The comparison gene set included only nonpseudogenes that were not identified as putative horizontally transferred genes.

### Microbial community analysis.

16S rRNA amplicon sequence data sets analyzed via Qiime (134) as described previously (24) were reanalyzed using Mothur v.1.40.3 (135). Reads shorter than 200 bp and reads containing ambiguous bases or homopolymeric runs longer than 7 bases were removed from the data set. Chimeric sequences were identified using VSEARCH (136) and removed from the data set. Reads were taxonomically classified against the Silva database (version 132), and all reads classified as unknown, eukaryotic, or mitochondrial or as representative of chloroplasts were removed from the data set. Reads were aligned using the Silva database (v. 132) as the reference and clustered into operational taxonomic units (OTUs) at a distance of 0.03, an approximation used for bacterial species. Counts of OTUs per sample were generated, and the top 10 most abundant OTUs were plotted (see Fig. S3 in the supplemental material). The top 50 most abundant OTUs were queried against the “nt” nucleotide database using BLAST for taxonomic classification.

### Data availability.

The Burkholderia gladioli Lv-StA genome was deposited in GenBank under accession number WITE00000000. The metagenomic bins obtained in this study were deposited in GenBank under the following accession numbers: for *Burkholderia* sp. Lv-StB, WNDN00000000; for DBSCAN_round1_2, WNDO00000000; for DBSCAN_round1_3, WNDP00000000; for DBSCAN_round2_3, WNDQ00000000; for DBSCAN_round34_1, WNDR00000000; for DBSCAN_round3_0, WNDS00000000; for DBSCAN_round43_4, WNDT00000000; for DBSCAN_round44_0, WNDU00000000; for DBSCAN_round4_0, WNDV00000000; for DBSCAN_round4_12, WNDW00000000; for DBSCAN_round4_6, WNDX00000000; for DBSCAN_round5_1, WNDY00000000; for DBSCAN_round5_3, WNDZ00000000; for DBSCAN_round6_14, WNEA00000000; for DBSCAN_round6_18, WNEB00000000; for DBSCAN_round7_0, WNEC00000000; for DBSCAN_round7_14, WNED00000000; for DBSCAN_round8_1, WNEE00000000. The raw Illumina reads used to construct the L. villosa egg metagenome were deposited to the SRA database under accession number PRJNA531449.

## References

[1] Shigenobu S, Watanabe H, Hattori M, Sakaki Y, Ishikawa H 2000 Genome sequence of the endocellular bacterial symbiont of aphids *Buchnera* sp. APS. Nature 407:81–86. doi:10.1038/35024074. 10993077

[2] Akman L, Yamashita A, Watanabe H, Oshima K, Shiba T, Hattori M, Aksoy S 2002 Genome sequence of the endocellular obligate symbiont of tsetse flies, *Wigglesworthia glossinidia*. Nat Genet 32:402–407. doi:10.1038/ng986. 12219091

[3] Miller IJ, Vanee N, Fong SS, Lim-Fong GE, Kwan JC 2016 Lack of overt genome reduction in the bryostatin-producing bryozoan symbiont “*Candidatus* Endobugula sertula.” Appl Environ Microbiol 82:6573–6583. doi:10.1128/AEM.01800-16. 27590822 PMC5086551

[4] Lopera J, Miller IJ, McPhail KL, Kwan JC 2017 Increased biosynthetic gene dosage in a genome-reduced defensive bacterial symbiont. mSystems 2:e00096-17. doi:10.1128/mSystems.00096-17. 29181447 PMC5698493

[5] Piel J 2002 A polyketide synthase-peptide synthetase gene cluster from an uncultured bacterial symbiont of *Paederus* beetles. Proc Natl Acad Sci U S A 99:14002–14007. doi:10.1073/pnas.222481399. 12381784 PMC137826

[6] Flórez LV, Scherlach K, Miller IJ, Rodrigues A, Kwan JC, Hertweck C, Kaltenpoth M 2018 An antifungal polyketide associated with horizontally acquired genes supports symbiont-mediated defense in *Lagria villosa* beetles. Nat Commun 9:2478. doi:10.1038/s41467-018-04955-6. 29946103 PMC6018673

[7] Kroiss J, Kaltenpoth M, Schneider B, Schwinger M-G, Hertweck C, Maddula RK, Strohm E, Svatos A 2010 Symbiotic *Streptomycetes* provide antibiotic combination prophylaxis for wasp offspring. Nat Chem Biol 6:261–263. doi:10.1038/nchembio.331. 20190763

[8] Currie CR, Scott JA, Summerbell RC, Malloch D 1999 Fungus-growing ants use antibiotic-producing bacteria to control garden parasites. Nature 398:701–704. doi:10.1038/19519.

[9] McCutcheon JP, Moran NA 2011 Extreme genome reduction in symbiotic bacteria. Nat Rev Microbiol 10:13–26. doi:10.1038/nrmicro2670. 22064560

[10] Latorre A, Manzano-Marín A 2017 Dissecting genome reduction and trait loss in insect endosymbionts. Ann N Y Acad Sci 1389:52–75. doi:10.1111/nyas.13222. 27723934

[11] Lo W-S, Huang Y-Y, Kuo C-H 2016 Winding paths to simplicity: genome evolution in facultative insect symbionts. FEMS Microbiol Rev 40:855–874. doi:10.1093/femsre/fuw028. 28204477 PMC5091035

[12] Shih PM, Matzke NJ 2013 Primary endosymbiosis events date to the later Proterozoic with cross-calibrated phylogenetic dating of duplicated ATPase proteins. Proc Natl Acad Sci U S A 110:12355–12360. doi:10.1073/pnas.1305813110. 23776247 PMC3725117

[13] López-García P, Eme L, Moreira D 2017 Symbiosis in eukaryotic evolution. J Theor Biol 434:20–33. doi:10.1016/j.jtbi.2017.02.031. 28254477 PMC5638015

[14] Munson MA, Baumann P, Clark MA, Baumann L, Moran NA, Voegtlin DJ, Campbell BC 1991 Evidence for the establishment of aphid-eubacterium endosymbiosis in an ancestor of four aphid families. J Bacteriol 173:6321–6324. doi:10.1128/jb.173.20.6321-6324.1991. 1917864 PMC208962

[15] Moran NA, Munson MA, Baumann P, Ishikawa H 1993 A molecular clock in endosymbiotic bacteria is calibrated using the insect hosts. Proc R Soc Lond B Biol Sci 253:167–171.

[16] Novichkov PS, Wolf YI, Dubchak I, Koonin EV 2009 Trends in prokaryotic evolution revealed by comparison of closely related bacterial and archaeal genomes. J Bacteriol 191:65–73. doi:10.1128/JB.01237-08. 18978059 PMC2612427

[17] Kuo C-H, Moran NA, Ochman H 2009 The consequences of genetic drift for bacterial genome complexity. Genome Res 19:1450–1454. doi:10.1101/gr.091785.109. 19502381 PMC2720180

[18] Mira A, Ochman H, Moran NA 2001 Deletional bias and the evolution of bacterial genomes. Trends Genet 17:589–596. doi:10.1016/s0168-9525(01)02447-7. 11585665

[19] Giovannoni SJ, Tripp HJ, Givan S, Podar M, Vergin KL, Baptista D, Bibbs L, Eads J, Richardson TH, Noordewier M, Rappé MS, Short JM, Carrington JC, Mathur EJ 2005 Genome streamlining in a cosmopolitan oceanic bacterium. Science 309:1242–1245. doi:10.1126/science.1114057. 16109880

[20] Dufresne A, Garczarek L, Partensky F 2005 Accelerated evolution associated with genome reduction in a free-living prokaryote. Genome Biol 6:R14. doi:10.1186/gb-2005-6-2-r14. 15693943 PMC551534

[21] Morris JJ, Lenski RE, Zinser ER 2012 The black queen hypothesis: evolution of dependencies through adaptive gene loss. mBio 3:e00036-12. doi:10.1128/mBio.00036-12. 22448042 PMC3315703

[22] Dietel A-K, Kaltenpoth M, Kost C 2018 Convergent evolution in intracellular elements: plasmids as model endosymbionts. Trends Microbiol 26:755–768. doi:10.1016/j.tim.2018.03.004. 29650391

[23] Dietel A-K, Merker H, Kaltenpoth M, Kost C 2019 Selective advantages favour high genomic AT-contents in intracellular elements. PLoS Genet 15:e1007778. doi:10.1371/journal.pgen.1007778. 31034469 PMC6519830

[24] Flórez LV, Scherlach K, Gaube P, Ross C, Sitte E, Hermes C, Rodrigues A, Hertweck C, Kaltenpoth M 2017 Antibiotic-producing symbionts dynamically transition between plant pathogenicity and insect-defensive mutualism. Nat Commun 8:15172. doi:10.1038/ncomms15172. 28452358 PMC5414355

[25] Dose B, Niehs SP, Scherlach K, Flórez LV, Kaltenpoth M, Hertweck C 2018 Unexpected bacterial origin of the antibiotic icosalide: two-tailed depsipeptide assembly in multifarious *Burkholderia* symbionts. ACS Chem Biol 13:2414–2420. doi:10.1021/acschembio.8b00600. 30160099

[26] Flórez LV, Kaltenpoth M 2017 Symbiont dynamics and strain diversity in the defensive mutualism between *Lagria* beetles and *Burkholderia*. Environ Microbiol 19:3674–3688. doi:10.1111/1462-2920.13868. 28752961

[27] Waterworth SC, Flórez LV, Rees ER, Hertweck C, Kaltenpoth M, Kwan JC 2019 Horizontal gene transfer to a defensive symbiont with a reduced genome amongst a multipartite beetle microbiome. bioRxiv doi:10.1101/780619. PMC7042692 32098813

[28] Miller IJ, Rees ER, Ross J, Miller I, Baxa J, Lopera J, Kerby RL, Rey FE, Kwan JC 2019 Autometa: automated extraction of microbial genomes from individual shotgun metagenomes. Nucleic Acids Res 47:e57. doi:10.1093/nar/gkz148. 30838416 PMC6547426

[29] Parks DH, Chuvochina M, Waite DW, Rinke C, Skarshewski A, Chaumeil P-A, Hugenholtz P 2018 A standardized bacterial taxonomy based on genome phylogeny substantially revises the tree of life. Nat Biotechnol 36:996–1004. doi:10.1038/nbt.4229. 30148503

[30] Goris J, Konstantinidis KT, Klappenbach JA, Coenye T, Vandamme P, Tiedje JM 2007 DNA–DNA hybridization values and their relationship to whole-genome sequence similarities. Int J Syst Evol Microbiol 57:81–91. doi:10.1099/ijs.0.64483-0. 17220447

[31] Rinke C, Schwientek P, Sczyrba A, Ivanova NN, Anderson IJ, Cheng J-F, Darling A, Malfatti S, Swan BK, Gies EA, Dodsworth JA, Hedlund BP, Tsiamis G, Sievert SM, Liu W-T, Eisen JA, Hallam SJ, Kyrpides NC, Stepanauskas R, Rubin EM, Hugenholtz P, Woyke T 2013 Insights into the phylogeny and coding potential of microbial dark matter. Nature 499:431–437. doi:10.1038/nature12352. 23851394

[32] Miller IJ, Weyna TR, Fong SS, Lim-Fong GE, Kwan JC 2016 Single sample resolution of rare microbial dark matter in a marine invertebrate metagenome. Sci Rep 6:34362. doi:10.1038/srep34362. 27681823 PMC5041132

[33] Miller IJ, Chevrette MG, Kwan JC 2017 Interpreting microbial biosynthesis in the genomic age: biological and practical considerations. Mar Drugs 15:165. doi:10.3390/md15060165. PMC5484115 28587290

[34] Blin K, Wolf T, Chevrette MG, Lu X, Schwalen CJ, Kautsar SA, Suarez Duran HG, de Los Santos ELC, Kim HU, Nave M, Dickschat JS, Mitchell DA, Shelest E, Breitling R, Takano E, Lee SY, Weber T, Medema MH 2017 antiSMASH 4.0-improvements in chemistry prediction and gene cluster boundary identification. Nucleic Acids Res 45:W36–W41. doi:10.1093/nar/gkx319. 28460038 PMC5570095

[35] Navarro-Muñoz JC, Selem-Mojica N, Mullowney MW, Kautsar SA, Tryon JH, Parkinson EI, De Los Santos ELC, Yeong M, Cruz-Morales P, Abubucker S, Roeters A, Lokhorst W, Fernandez-Guerra A, Cappelini LTD, Goering AW, Thomson RJ, Metcalf WW, Kelleher NL, Barona-Gomez F, Medema MH 2020 A computational framework to explore large-scale biosynthetic diversity. Nat Chem Biol 16:60–68. doi:10.1038/s41589-019-0400-9. 31768033 PMC6917865

[36] Silva FJ, Santos-Garcia D 2015 Slow and fast evolving endosymbiont lineages: positive correlation between the rates of synonymous and non-synonymous substitution. Front Microbiol 6:1279. doi:10.3389/fmicb.2015.01279. 26617602 PMC4643148

[37] Altenhoff AM, Glover NM, Train C-M, Kaleb K, Warwick Vesztrocy A, Dylus D, de Farias TM, Zile K, Stevenson C, Long J, Redestig H, Gonnet GH, Dessimoz C 2018 The OMA orthology database in 2018: retrieving evolutionary relationships among all domains of life through richer Web and programmatic interfaces. Nucleic Acids Res 46:D477–D485. doi:10.1093/nar/gkx1019. 29106550 PMC5753216

[38] Dessimoz C, Cannarozzi G, Gil M, Margadant D, Roth A, Schneider A, Gonnet GH 2005 OMA, a comprehensive, automated project for the identification of orthologs from complete genome data: introduction and first achievements, p 61–72. *In* Comparative genomics. Springer, Berlin, Germany.

[39] Page AJ, Cummins CA, Hunt M, Wong VK, Reuter S, Holden MTG, Fookes M, Falush D, Keane JA, Parkhill J 2015 Roary: rapid large-scale prokaryote pan genome analysis. Bioinformatics 31:3691–3693. doi:10.1093/bioinformatics/btv421. 26198102 PMC4817141

[40] Lin J-J, Sancar A 1992 Active site of (A) BC excinuclease. I. Evidence for 5′ incision by UvrC through a catalytic site involving Asp399, Asp438, Asp466, and His538 residues. J Biol Chem 267:17688–17692. 1387639

[41] Carter AT, Pearson BM, Dickinson JR, Lancashire WE 1993 Sequence of the *Escherichia coli* K-12 *edd* and *eda* genes of the Entner-Doudoroff pathway. Gene 130:155–156. doi:10.1016/0378-1119(93)90362-7. 8344525

[42] Lunin VV, Li Y, Schrag JD, Iannuzzi P, Cygler M, Matte A 2004 Crystal structures of *Escherichia coli* ATP-dependent glucokinase and its complex with glucose. J Bacteriol 186:6915–6927. doi:10.1128/JB.186.20.6915-6927.2004. 15466045 PMC522197

[43] Almassy RJ, Janson CA, Kan CC, Hostomska Z 1992 Structures of apo and complexed *Escherichia coli* glycinamide ribonucleotide transformylase. Proc Natl Acad Sci U S A 89:6114–6118. doi:10.1073/pnas.89.13.6114. 1631098 PMC49448

[44] Schendel FJ, Mueller E, Stubbe J, Shiau A, Smith JM 1989 Formylglycinamide ribonucleotide synthetase from *Escherichia coli*: cloning, sequencing, overproduction, isolation, and characterization. Biochemistry 28:2459–2471. doi:10.1021/bi00432a017. 2659070

[45] Tchigvintsev A, Tchigvintsev D, Flick R, Popovic A, Dong A, Xu X, Brown G, Lu W, Wu H, Cui H, Dombrowski L, Joo JC, Beloglazova N, Min J, Savchenko A, Caudy AA, Rabinowitz JD, Murzin AG, Yakunin AF 2013 Biochemical and structural studies of conserved Maf proteins revealed nucleotide pyrophosphatases with a preference for modified nucleotides. Chem Biol 20:1386–1398. doi:10.1016/j.chembiol.2013.09.011. 24210219 PMC3899018

[46] Jin J, Wu R, Zhu J, Yang S, Lei Z, Wang N, Singh VK, Zheng J, Jia Z 2015 Insights into the cellular function of YhdE, a nucleotide pyrophosphatase from *Escherichia coli*. PLoS One 10:e0117823. doi:10.1371/journal.pone.0117823. 25658941 PMC4319933

[47] Boël G, Smith PC, Ning W, Englander MT, Chen B, Hashem Y, Testa AJ, Fischer JJ, Wieden H-J, Frank J, Gonzalez RL Jr, Hunt JF 2014 The ABC-F protein EttA gates ribosome entry into the translation elongation cycle. Nat Struct Mol Biol 21:143–151. doi:10.1038/nsmb.2740. 24389466 PMC4101993

[48] Chen B, Boël G, Hashem Y, Ning W, Fei J, Wang C, Gonzalez RL Jr, Hunt JF, Frank J 2014 EttA regulates translation by binding the ribosomal E site and restricting ribosome-tRNA dynamics. Nat Struct Mol Biol 21:152–159. doi:10.1038/nsmb.2741. 24389465 PMC4143144

[49] Dolan SK, Welch M 2018 The glyoxylate shunt, 60 years on. Annu Rev Microbiol 72:309–330. doi:10.1146/annurev-micro-090817-062257. 30200852

[50] Raghunathan D, Wells TJ, Morris FC, Shaw RK, Bobat S, Peters SE, Paterson GK, Jensen KT, Leyton DL, Blair JMA, Browning DF, Pravin J, Flores-Langarica A, Hitchcock JR, Moraes CTP, Piazza RMF, Maskell DJ, Webber MA, May RC, MacLennan CA, Piddock LJ, Cunningham AF, Henderson IR 2011 SadA, a trimeric autotransporter from *Salmonella enterica* serovar Typhimurium, can promote biofilm formation and provides limited protection against infection. Infect Immun 79:4342–4352. doi:10.1128/IAI.05592-11. 21859856 PMC3257908

[51] Linke D, Riess T, Autenrieth IB, Lupas A, Kempf V 2006 Trimeric autotransporter adhesins: variable structure, common function. Trends Microbiol 14:264–270. doi:10.1016/j.tim.2006.04.005. 16678419

[52] Powell JE, Leonard SP, Kwong WK, Engel P, Moran NA 2016 Genome-wide screen identifies host colonization determinants in a bacterial gut symbiont. Proc Natl Acad Sci U S A 113:13887–13892. doi:10.1073/pnas.1610856113. 27849596 PMC5137728

[53] Kwong WK, Engel P, Koch H, Moran NA 2014 Genomics and host specialization of honey bee and bumble bee gut symbionts. Proc Natl Acad Sci U S A 111:11509–11514. doi:10.1073/pnas.1405838111. 25053814 PMC4128107

[54] Chen X, Schreiber K, Appel J, Makowka A, Fähnrich B, Roettger M, Hajirezaei MR, Sönnichsen FD, Schönheit P, Martin WF, Gutekunst K 2016 The Entner-Doudoroff pathway is an overlooked glycolytic route in cyanobacteria and plants. Proc Natl Acad Sci U S A 113:5441–5446. doi:10.1073/pnas.1521916113. 27114545 PMC4868481

[55] Takeya M, Hirai MY, Osanai T 2017 Allosteric inhibition of phosphoenolpyruvate carboxylases is determined by a single amino acid residue in cyanobacteria. Sci Rep 7:41080. doi:10.1038/srep41080. 28117365 PMC5259782

[56] Duarte M, Videira A 2009 Effects of mitochondrial complex III disruption in the respiratory chain of *Neurospora crassa*. Mol Microbiol 72:246–258. doi:10.1111/j.1365-2958.2009.06643.x. 19239619

[57] Jain M, Munoz-Bodnar A, Gabriel DW 2017 Concomitant loss of the glyoxalase system and glycolysis makes the uncultured pathogen “*Candidatus* Liberibacter asiaticus” an energy scavenger. Appl Environ Microbiol 83:e01670-17. doi:10.1128/AEM.01670-17. 28939611 PMC5691416

[58] Porter TN, Li Y, Raushel FM 2004 Mechanism of the dihydroorotase reaction. Biochemistry 43:16285–16292. doi:10.1021/bi048308g. 15610022

[59] Aghajari N, Jensen KF, Gajhede M 1994 Crystallization and preliminary X-ray diffraction studies on the apo form of orotate phosphoribosyltransferase from *Escherichia coli*. J Mol Biol 241:292–294. doi:10.1006/jmbi.1994.1503. 8057372

[60] Brignole EJ, Ando N, Zimanyi CM, Drennan CL 2012 The prototypic class Ia ribonucleotide reductase from *Escherichia coli*: still surprising after all these years. Biochem Soc Trans 40:523–530. doi:10.1042/BST20120081. 22616862 PMC5912335

[61] Carreras CW, Santi DV 1995 The catalytic mechanism and structure of thymidylate synthase. Annu Rev Biochem 64:721–762. doi:10.1146/annurev.bi.64.070195.003445. 7574499

[62] Nelson DL, Lehninger AL, Cox MM 2008 Lehninger principles of biochemistry. W. H. Freeman, New York, NY.

[63] Feng H, Edwards N, Anderson CMH, Althaus M, Duncan RP, Hsu Y-C, Luetje CW, Price DRG, Wilson ACC, Thwaites DT 2019 Trading amino acids at the aphid-*Buchnera* symbiotic interface. Proc Natl Acad Sci U S A 116:16003–16011. doi:10.1073/pnas.1906223116. 31337682 PMC6690024

[64] Gerdes SY, Scholle MD, Campbell JW, Balázsi G, Ravasz E, Daugherty MD, Somera AL, Kyrpides NC, Anderson I, Gelfand MS, Bhattacharya A, Kapatral V, D'Souza M, Baev MV, Grechkin Y, Mseeh F, Fonstein MY, Overbeek R, Barabási A-L, Oltvai ZN, Osterman AL 2003 Experimental determination and system level analysis of essential genes in *Escherichia coli* MG1655. J Bacteriol 185:5673–5684. doi:10.1128/jb.185.19.5673-5684.2003. 13129938 PMC193955

[65] Goodall ECA, Robinson A, Johnston IG, Jabbari S, Turner KA, Cunningham AF, Lund PA, Cole JA, Henderson IR 2018 The essential genome of *Escherichia coli* K-12. mBio 9:e02096-17. doi:10.1128/mBio.02096-17. 29463657 PMC5821084

[66] Moule MG, Hemsley CM, Seet Q, Guerra-Assunção JA, Lim J, Sarkar-Tyson M, Clark TG, Tan PBO, Titball RW, Cuccui J, Wren BW 2014 Genome-wide saturation mutagenesis of *Burkholderia pseudomallei* K96243 predicts essential genes and novel targets for antimicrobial development. mBio 5:e00926-13. doi:10.1128/mBio.00926-13. 24520057 PMC3950516

[67] Higgins S, Sanchez-Contreras M, Gualdi S, Pinto-Carbó M, Carlier A, Eberl L 2017 The essential genome of *Burkholderia cenocepacia* H111. J Bacteriol 199:e00260-17. doi:10.1128/JB.00260-17. 28847919 PMC5648868

[68] Kowalczykowski SC, Dixon DA, Eggleston AK, Lauder SD, Rehrauer WM 1994 Biochemistry of homologous recombination in *Escherichia coli*. Microbiol Rev 58:401–465. 7968921 10.1128/mr.58.3.401-465.1994 PMC372975

[69] Pitcher RS, Brissett NC, Doherty AJ 2007 Nonhomologous end-joining in bacteria: a microbial perspective. Annu Rev Microbiol 61:259–282. doi:10.1146/annurev.micro.61.080706.093354. 17506672

[70] McCullough AK, Dodson ML, Lloyd RS 1999 Initiation of base excision repair: glycosylase mechanisms and structures. Annu Rev Biochem 68:255–285. doi:10.1146/annurev.biochem.68.1.255. 10872450

[71] Boiteux S, Gajewski E, Laval J, Dizdaroglu M 1992 Substrate specificity of the *Escherichia coli* Fpg protein formamidopyrimidine-DNA glycosylase: excision of purine lesions in DNA produced by ionizing radiation or photosensitization. Biochemistry 31:106–110. doi:10.1021/bi00116a016. 1731864

[72] Barrett TE, Savva R, Panayotou G, Barlow T, Brown T, Jiricny J, Pearl LH 1998 Crystal structure of a G:T/U mismatch-specific DNA glycosylase: mismatch recognition by complementary-strand interactions. Cell 92:117–129. doi:10.1016/S0092-8674(00)80904-6. 9489705

[73] Saparbaev M, Laval J 1998 3,*N*^4^-ethenocytosine, a highly mutagenic adduct, is a primary substrate for *Escherichia coli* double-stranded uracil-DNA glycosylase and human mismatch-specific thymine-DNA glycosylase. Proc Natl Acad Sci U S A 95:8508–8513. doi:10.1073/pnas.95.15.8508. 9671708 PMC21106

[74] Hang B, Downing G, Guliaev AB, Singer B 2002 Novel activity of *Escherichia coli* mismatch uracil-DNA glycosylase (Mug) excising 8-(hydroxymethyl)-3,*N*^4^-ethenocytosine, a potential product resulting from glycidaldehyde reaction. Biochemistry 41:2158–2165. doi:10.1021/bi011542b. 11841206

[75] Saparbaev M, Langouët S, Privezentzev CV, Guengerich FP, Cai H, Elder RH, Laval J 2002 1,*N*^2^-Ethenoguanine, a mutagenic DNA adduct, is a primary substrate of *Escherichia coli* mismatch-specific uracil-DNA glycosylase and human alkylpurine-DNA-*N*-glycosylase. J Biol Chem 277:26987–26993. doi:10.1074/jbc.M111100200. 12016206

[76] Liu P, Burdzy A, Sowers LC 2003 Repair of the mutagenic DNA oxidation product, 5-formyluracil. DNA Repair (Amst) 2:199–210. doi:10.1016/s1568-7864(02)00198-2. 12531390

[77] Schofield MJ, Hsieh P 2003 DNA mismatch repair: molecular mechanisms and biological function. Annu Rev Microbiol 57:579–608. doi:10.1146/annurev.micro.57.030502.090847. 14527292

[78] Gillings MR 2017 Lateral gene transfer, bacterial genome evolution, and the Anthropocene. Ann N Y Acad Sci 1389:20–36. doi:10.1111/nyas.13213. 27706829

[79] Mathew GM, Ju Y-M, Lai C-Y, Mathew DC, Huang CC 2012 Microbial community analysis in the termite gut and fungus comb of *Odontotermes formosanus*: the implication of *Bacillus* as mutualists. FEMS Microbiol Ecol 79:504–517. doi:10.1111/j.1574-6941.2011.01232.x. 22092951

[80] Jones AG, Mason CJ, Felton GW, Hoover K 2019 Host plant and population source drive diversity of microbial gut communities in two polyphagous insects. Sci Rep 9:2792. doi:10.1038/s41598-019-39163-9. 30808905 PMC6391413

[81] Montagna M, Chouaia B, Mazza G, Prosdocimi EM, Crotti E, Mereghetti V, Vacchini V, Giorgi A, De Biase A, Longo S, Cervo R, Lozzia GC, Alma A, Bandi C, Daffonchio D 2015 Effects of the diet on the microbiota of the red palm weevil (Coleoptera: Dryophthoridae). PLoS One 10:e0117439. doi:10.1371/journal.pone.0117439. 25635833 PMC4311986

[82] McClure EA, Nelson MC, Lin A, Graf J 2019 *Macrobdella decora*: Old World leech gut microbial community structure conserved in a new world leech. bioRxiv doi:10.1101/687418. PMC8117757 33674439

[83] Rio RVM, Maltz M, McCormick B, Reiss A, Graf J 2009 Symbiont succession during embryonic development of the European medicinal leech, *Hirudo verbana*. Appl Environ Microbiol 75:6890–6895. doi:10.1128/AEM.01129-09. 19648363 PMC2772434

[84] Chen EZ, Bushman FD, Li H 2017 A model-based approach for species abundance quantification based on shotgun metagenomic data. Stat Biosci 9:13–27. doi:10.1007/s12561-016-9148-x. 28959368 PMC5612490

[85] Delforno TP, Lacerda Júnior GV, Noronha MF, Sakamoto IK, Varesche MBA, Oliveira VM 2017 Microbial diversity of a full-scale UASB reactor applied to poultry slaughterhouse wastewater treatment: integration of 16S rRNA gene amplicon and shotgun metagenomic sequencing. MicrobiologyOpen 6:e443. doi:10.1002/mbo3.443. PMC5458456 28229558

[86] Becq J, Churlaud C, Deschavanne P 2010 A benchmark of parametric methods for horizontal transfers detection. PLoS One 5:e9989. doi:10.1371/journal.pone.0009989. 20376325 PMC2848678

[87] Lawrence JG, Ochman H 1997 Amelioration of bacterial genomes: rates of change and exchange. J Mol Evol 44:383–397. doi:10.1007/pl00006158. 9089078

[88] Muto A, Osawa S 1987 The guanine and cytosine content of genomic DNA and bacterial evolution. Proc Natl Acad Sci U S A 84:166–169. doi:10.1073/pnas.84.1.166. 3467347 PMC304163

[89] Jensen PR 2016 Natural products and the gene cluster revolution. Trends Microbiol 24:968–977. doi:10.1016/j.tim.2016.07.006. 27491886 PMC5123934

[90] Bauer E, Kaltenpoth M, Salem H 3 12 2019, posting date Minimal fermentative metabolism fuels extracellular symbiont in a leaf beetle. ISME J doi:10.1038/s41396-019-0562-1. PMC7031334 31796934

[91] Mohni KN, Wessel SR, Zhao R, Wojciechowski AC, Luzwick JW, Layden H, Eichman BF, Thompson PS, Mehta KPM, Cortez D 2019 HMCES maintains genome integrity by shielding abasic sites in single-strand DNA. Cell 176:144–153.e13. doi:10.1016/j.cell.2018.10.055. 30554877 PMC6329640

[92] Di Pierro M, Lu R, Uzzau S, Wang W, Margaretten K, Pazzani C, Maimone F, Fasano A 2001 Zonula occludens toxin structure-function analysis. Identification of the fragment biologically active on tight junctions and of the zonulin receptor binding domain. J Biol Chem 276:19160–19165. doi:10.1074/jbc.M009674200. 11278543

[93] Szklarczyk D, Morris JH, Cook H, Kuhn M, Wyder S, Simonovic M, Santos A, Doncheva NT, Roth A, Bork P, Jensen LJ, von Mering C 2017 The STRING database in 2017: quality-controlled protein-protein association networks, made broadly accessible. Nucleic Acids Res 45:D362–D368. doi:10.1093/nar/gkw937. 27924014 PMC5210637

[94] Liu F, Lee H, Lan R, Zhang L 2016 Zonula occludens toxins and their prophages in *Campylobacter* species. Gut Pathog 8:43. doi:10.1186/s13099-016-0125-1. 27651834 PMC5025632

[95] Mahendran V, Liu F, Riordan SM, Grimm MC, Tanaka MM, Zhang L 2016 Examination of the effects of *Campylobacter concisus* zonula occludens toxin on intestinal epithelial cells and macrophages. Gut Pathog 8:18. doi:10.1186/s13099-016-0101-9. 27195022 PMC4870807

[96] Joseph B, Schwarz RF, Linke B, Blom J, Becker A, Claus H, Goesmann A, Frosch M, Müller T, Vogel U, Schoen C 2011 Virulence evolution of the human pathogen *Neisseria meningitidis* by recombination in the core and accessory genome. PLoS One 6:e18441. doi:10.1371/journal.pone.0018441. 21541312 PMC3082526

[97] Stammer HJ 1929 Die Symbiose der Lagriiden (Coleoptera). Zoomorphology 15:1–34.

[98] Kumar S, Stecher G, Suleski M, Hedges SB 2017 TimeTree: a resource for timelines, timetrees, and divergence times. Mol Biol Evol 34:1812–1819. doi:10.1093/molbev/msx116. 28387841

[99] Kergoat GJ, Bouchard P, Clamens A-L, Abbate JL, Jourdan H, Jabbour-Zahab R, Genson G, Soldati L, Condamine FL 2014 Cretaceous environmental changes led to high extinction rates in a hyperdiverse beetle family. BMC Evol Biol 14:220. doi:10.1186/s12862-014-0220-1. 25331733 PMC4210489

[100] Kwan JC, Donia MS, Han AW, Hirose E, Haygood MG, Schmidt EW 2012 Genome streamlining and chemical defense in a coral reef symbiosis. Proc Natl Acad Sci U S A 109:20655–20660. doi:10.1073/pnas.1213820109. 23185008 PMC3528492

[101] Kwan JC, Schmidt EW 2013 Bacterial endosymbiosis in a chordate host: long-term co-evolution and conservation of secondary metabolism. PLoS One 8:e80822. doi:10.1371/journal.pone.0080822. 24324632 PMC3851785

[102] Pinto-Carbó M, Gademann K, Eberl L, Carlier A 2018 Leaf nodule symbiosis: function and transmission of obligate bacterial endophytes. Curr Opin Plant Biol 44:23–31. doi:10.1016/j.pbi.2018.01.001. 29452904

[103] Carlier A, Fehr L, Pinto-Carbó M, Schäberle T, Reher R, Dessein S, König G, Eberl L 2016 The genome analysis of *Candidatus* Burkholderia crenata reveals that secondary metabolism may be a key function of the *Ardisia crenata* leaf nodule symbiosis. Environ Microbiol 18:2507–2522. doi:10.1111/1462-2920.13184. 26663534

[104] Pinto-Carbó M, Sieber S, Dessein S, Wicker T, Verstraete B, Gademann K, Eberl L, Carlier A 2016 Evidence of horizontal gene transfer between obligate leaf nodule symbionts. ISME J 10:2092–2105. doi:10.1038/ismej.2016.27. 26978165 PMC4989318

[105] Flórez LV, Biedermann PHW, Engl T, Kaltenpoth M 2015 Defensive symbioses of animals with prokaryotic and eukaryotic microorganisms. Nat Prod Rep 32:904–936. doi:10.1039/c5np00010f. 25891201

[106] Salem H, Bauer E, Kirsch R, Berasategui A, Cripps M, Weiss B, Koga R, Fukumori K, Vogel H, Fukatsu T, Kaltenpoth M 2017 Drastic genome reduction in an herbivore’s pectinolytic symbiont. Cell 171:1520–1531.e13. doi:10.1016/j.cell.2017.10.029. 29153832

[107] Kenyon LJ, Meulia T, Sabree ZL 2015 Habitat visualization and genomic analysis of “*Candidatus* Pantoea carbekii,” the primary symbiont of the brown marmorated stink bug. Genome Biol Evol 7:620–635. doi:10.1093/gbe/evv006. 25587021 PMC4350177

[108] Nikoh N, Hosokawa T, Oshima K, Hattori M, Fukatsu T 2011 Reductive evolution of bacterial genome in insect gut environment. Genome Biol Evol 3:702–714. doi:10.1093/gbe/evr064. 21737395 PMC3157840

[109] Kikuchi Y, Hosokawa T, Nikoh N, Meng X-Y, Kamagata Y, Fukatsu T 2009 Host-symbiont co-speciation and reductive genome evolution in gut symbiotic bacteria of acanthosomatid stinkbugs. BMC Biol 7:2. doi:10.1186/1741-7007-7-2. 19146674 PMC2637841

[110] Kaiwa N, Hosokawa T, Nikoh N, Tanahashi M, Moriyama M, Meng X-Y, Maeda T, Yamaguchi K, Shigenobu S, Ito M, Fukatsu T 2014 Symbiont-supplemented maternal investment underpinning host’s ecological adaptation. Curr Biol 24:2465–2470. doi:10.1016/j.cub.2014.08.065. 25264255

[111] Pombert J-F, Selman M, Burki F, Bardell FT, Farinelli L, Solter LF, Whitman DW, Weiss LM, Corradi N, Keeling PJ 2012 Gain and loss of multiple functionally related, horizontally transferred genes in the reduced genomes of two microsporidian parasites. Proc Natl Acad Sci U S A 109:12638–12643. doi:10.1073/pnas.1205020109. 22802648 PMC3412028

[112] Brown BP, Wernegreen JJ 2019 Genomic erosion and extensive horizontal gene transfer in gut-associated Acetobacteraceae. BMC Genomics 20:472. doi:10.1186/s12864-019-5844-5. 31182035 PMC6558740

[113] Harmer CJ, Hall RM 2016 IS*26*-mediated formation of transposons carrying antibiotic resistance genes. mSphere 1:e00038-16. doi:10.1128/mSphere.00038-16. PMC4894685 27303727

[114] Zupancic TJ, Marvo SL, Chung JH, Peralta EG, Jaskunas SR 1983 RecA-independent recombination between direct repeats of IS50. Cell 33:629–637. doi:10.1016/0092-8674(83)90444-0. 6345001

[115] Metcalf JA, Bordenstein SR 2012 The complexity of virus systems: the case of endosymbionts. Curr Opin Microbiol 15:546–552. doi:10.1016/j.mib.2012.04.010. 22609369 PMC3424318

[116] Bennett GM, Moran NA 2015 Heritable symbiosis: the advantages and perils of an evolutionary rabbit hole. Proc Natl Acad Sci U S A 112:10169–10176. doi:10.1073/pnas.1421388112. 25713367 PMC4547261

[117] Moran NA 1996 Accelerated evolution and Muller’s rachet in endosymbiotic bacteria. Proc Natl Acad Sci U S A 93:2873–2878. doi:10.1073/pnas.93.7.2873. 8610134 PMC39726

[118] Ondov BD, Treangen TJ, Melsted P, Mallonee AB, Bergman NH, Koren S, Phillippy AM 2016 Mash: fast genome and metagenome distance estimation using MinHash. Genome Biol 17:132. doi:10.1186/s13059-016-0997-x. 27323842 PMC4915045

[119] Seemann T 2014 Prokka: rapid prokaryotic genome annotation. Bioinformatics 30:2068–2069. doi:10.1093/bioinformatics/btu153. 24642063

[120] Buchfink B, Xie C, Huson DH 2015 Fast and sensitive protein alignment using DIAMOND. Nat Methods 12:59–60. doi:10.1038/nmeth.3176. 25402007

[121] Lerat E, Ochman H 2005 Recognizing the pseudogenes in bacterial genomes. Nucleic Acids Res 33:3125–3132. doi:10.1093/nar/gki631. 15933207 PMC1142405

[122] Blin K, Shaw S, Steinke K, Villebro R, Ziemert N, Lee SY, Medema MH, Weber T 2019 antiSMASH 5.0: updates to the secondary metabolite genome mining pipeline. Nucleic Acids Res 47:W81–W87. doi:10.1093/nar/gkz310. 31032519 PMC6602434

[123] Alanjary M, Steinke K, Ziemert N 2019 AutoMLST: an automated Web server for generating multi-locus species trees highlighting natural product potential. Nucleic Acids Res 47:W276–W282. doi:10.1093/nar/gkz282. 30997504 PMC6602446

[124] Jain C, Rodriguez-R LM, Phillippy AM, Konstantinidis KT, Aluru S 2018 High throughput ANI analysis of 90K prokaryotic genomes reveals clear species boundaries. Nat Commun 9:5114. doi:10.1038/s41467-018-07641-9. 30504855 PMC6269478

[125] Edgar RC 2004 MUSCLE: multiple sequence alignment with high accuracy and high throughput. Nucleic Acids Res 32:1792–1797. doi:10.1093/nar/gkh340. 15034147 PMC390337

[126] Suyama M, Torrents D, Bork P 2006 PAL2NAL: robust conversion of protein sequence alignments into the corresponding codon alignments. Nucleic Acids Res 34:W609–W612. doi:10.1093/nar/gkl315. 16845082 PMC1538804

[127] Yang Z 2007 PAML 4: phylogenetic analysis by maximum likelihood. Mol Biol Evol 24:1586–1591. doi:10.1093/molbev/msm088. 17483113

[128] Yang Z 2014 Molecular evolution: a statistical approach. OUP, Oxford, United Kingdom.

[129] Price MN, Dehal PS, Arkin AP 2010 FastTree 2–approximately maximum-likelihood trees for large alignments. PLoS One 5:e9490. doi:10.1371/journal.pone.0009490. 20224823 PMC2835736

[130] Kanehisa M, Sato Y, Furumichi M, Morishima K, Tanabe M 2019 New approach for understanding genome variations in KEGG. Nucleic Acids Res 47:D590–D595. doi:10.1093/nar/gky962. 30321428 PMC6324070

[131] Kanehisa M, Goto S 2000 KEGG: Kyoto encyclopedia of genes and genomes. Nucleic Acids Res 28:27–30. doi:10.1093/nar/28.1.27. 10592173 PMC102409

[132] Aramaki T, Blanc-Mathieu R, Endo H, Ohkubo K, Kanehisa M, Goto S, Ogata H 2019 KofamKOALA: KEGG ortholog assignment based on profile HMM and adaptive score threshold. bioRxiv doi:10.1101/602110. PMC7141845 31742321

[133] Graham ED, Heidelberg JF, Tully BJ 2018 Potential for primary productivity in a globally-distributed bacterial phototroph. ISME J 12:1861–1866. doi:10.1038/s41396-018-0091-3. 29523891 PMC6018677

[134] Caporaso JG, Kuczynski J, Stombaugh J, Bittinger K, Bushman FD, Costello EK, Fierer N, Peña AG, Goodrich JK, Gordon JI, Huttley GA, Kelley ST, Knights D, Koenig JE, Ley RE, Lozupone CA, McDonald D, Muegge BD, Pirrung M, Reeder J, Sevinsky JR, Turnbaugh PJ, Walters WA, Widmann J, Yatsunenko T, Zaneveld J, Knight R 2010 QIIME allows analysis of high-throughput community sequencing data. Nat Methods 7:335–336. doi:10.1038/nmeth.f.303. 20383131 PMC3156573

[135] Schloss PD, Westcott SL, Ryabin T, Hall JR, Hartmann M, Hollister EB, Lesniewski RA, Oakley BB, Parks DH, Robinson CJ, Sahl JW, Stres B, Thallinger GG, Van Horn DJ, Weber CF 2009 Introducing Mothur: open-source, platform-independent, community-supported software for describing and comparing microbial communities. Appl Environ Microbiol 75:7537–7541. doi:10.1128/AEM.01541-09. 19801464 PMC2786419

[136] Rognes T, Flouri T, Nichols B, Quince C, Mahé F 2016 VSEARCH: a versatile open source tool for metagenomics. PeerJ 4:e2584. doi:10.7717/peerj.2584. 27781170 PMC5075697

