# Supplementary material for: Horizontal Gene Transfer to a Defensive Symbiont with a Reduced Genome in a Multipartite Beetle Microbiome
Source: mBio. 2020 Feb 25;11(1):e02430-19. doi: 10.1128/mBio.02430-19 (PMC7042692; doi:10.1128/mBio.02430-19)
Supplement: TABLE S1 [file mBio.02430-19-st001.docx]

**Table S1.** Divergence rates used in this study (taken from Silva and Santos-Garcia 2015 [(30)](https://paperpile.com/c/uf9zAd/pmD3f)).

| Abbreviation | Symbiont | Host | dS/t | dN/t |
| --- | --- | --- | --- | --- |
| BFL | *Blochmannia floridans* | *Camponotus floridans* | 8.9 × 10^-8^ | 7.9 × 10^-9^ |
| BPN | *Blochmannia pennsylvanicus* | *Camponotus pennsylvanicus* | 6.4 × 10^-8^ | 5.6 × 10^-9^ |
| BOB | *Blochmannia obliquus* | *Colobopsis obliquus* | 7.9 ×10^-8^ | 5.2 × 10^-9^ |
| BAU | *Baumannia cicadellinicola* | *Graphocephala atropunctata, Homalodisca vitripennis* | 1.1 × 10^-8^ | 1.0 × 10^-9^ |
