# Supplementary material for: Horizontal Gene Transfer to a Defensive Symbiont with a Reduced Genome in a Multipartite Beetle Microbiome
Source: mBio. 2020 Feb 25;11(1):e02430-19. doi: 10.1128/mBio.02430-19 (PMC7042692; doi:10.1128/mBio.02430-19)
Supplement: TABLE S2 [file mBio.02430-19-st002.docx]

**Table S2.** Intact transporter genes identified in the *Burkholderia* sp. Lv-StB genome.

| Locus tag | BLAST Annotation [Organism] | Identity (%) | E value |
| --- | --- | --- | --- |
| E5299_01756 | ABC transporter [*Burkholderia gladioli*] | 89 | 1.20E-90 |
| E5299_01753 | ABC transporter ATP-binding protein [*Burkholderia gladioli*] | 92.1 | 1.10E-140 |
| E5299_01889 | ABC transporter ATP-binding protein [*Burkholderia gladioli*] | 93.8 | 0.00E+00 |
| E5299_01490 | ABC transporter ATP-binding protein [*Burkholderia gladioli*] | 92.3 | 7.20E-173 |
| E5299_01730 | ABC transporter ATP-binding protein [*Burkholderia* sp. A1] | 94.5 | 2.90E-114 |
| E5299_01489 | ABC transporter ATP-binding protein [*Burkholderia* sp. A1] | 89.7 | 2.70E-164 |
| E5299_00810 | ABC transporter ATP-binding protein [*Burkholderia*] | 93.7 | 6.50E-140 |
| E5299_00727 | ABC transporter ATP-binding protein/permease [*Burkholderia gladioli*] | 87.7 | 4.60E-295 |
| E5299_01696 | ABC transporter permease [*Burkholderia gladioli*] | 91.7 | 3.20E-140 |
| E5299_00882 | ABC transporter permease [*Burkholderia gladioli*] | 92.1 | 3.70E-184 |
| E5299_01752 | ABC transporter permease [*Burkholderia* sp. A1] | 89.1 | 3.80E-186 |
| E5299_00794 | ABC transporter permease [*Burkholderia*] | 94.4 | 4.00E-100 |
| E5299_00884 | ABC transporter permease subunit [*Burkholderia gladioli*] | 93.1 | 3.00E-174 |
| E5299_01347 | ABC transporter permease subunit [*Burkholderia gladioli*] | 92.6 | 1.60E-305 |
| E5299_01488 | ABC transporter permease subunit [*Burkholderia gladioli*] | 88.7 | 3.00E-146 |
| E5299_01487 | ABC transporter permease subunit [*Burkholderia*] | 89.9 | 1.50E-157 |
| E5299_00461 | ABC transporter substrate-binding protein [*Burkholderia gladioli*] | 93.1 | 9.30E-287 |
| E5299_00885 | ABC transporter substrate-binding protein [*Burkholderia gladioli*] | 90.7 | 0.00E+00 |
| E5299_00930 | ABC transporter substrate-binding protein [*Burkholderia gladioli*] | 87.2 | 9.60E-157 |
| E5299_00814 | ABC transporter substrate-binding protein [*Burkholderia*] | 92.3 | 1.00E-103 |
| E5299_02444 | BMP family ABC transporter substrate-binding protein [*Ochrobactrum pseudogrignonense*] | 99.4 | 1.70E-204 |
| E5299_01763 | carbohydrate ABC transporter permease [*Ochrobactrum pseudogrignonense*] | 98.9 | 9.90E-144 |
| E5299_00923 | cation ABC transporter substrate-binding protein [*Burkholderia gladioli*] | 89.5 | 1.90E-147 |
| E5299_00811 | lipid asymmetry maintenance ABC transporter permease subunit MlaE [*Burkholderia*] | 91.4 | 4.50E-119 |
| E5299_00106 | lipoprotein ABC transporter ATP-binding protein [*Burkholderia gladioli*] | 92.2 | 1.70E-112 |
| E5299_00105 | lipoprotein-releasing ABC transporter permease subunit [*Burkholderia*] | 94 | 5.70E-212 |
| E5299_00793 | methionine ABC transporter ATP-binding protein [*Burkholderia gladioli*] | 91.3 | 7.10E-168 |
| E5299_00881 | microcin ABC transporter ATP-binding protein [*Burkholderia gladioli*] | 87.6 | 2.30E-275 |
| E5299_01411 | nitrate/sulfonate/bicarbonate ABC transporter ATP-binding protein [*Burkholderia gladioli*] | 96.6 | 7.40E-242 |
| E5299_01729 | sugar ABC transporter permease [*Burkholderia* sp. A1] | 95.1 | 1.00E-137 |
| E5299_01761 | sugar ABC transporter permease [*Ochrobactrum*] | 82.6 | 3.40E-106 |
| E5299_01760 | sugar ABC transporter substrate-binding protein [*Ochrobactrum*] | 98.8 | 1.50E-239 |
| E5299_01639 | biopolymer transporter ExbD [*Burkholderia gladioli*] | 88.5 | 1.50E-60 |
| E5299_01407 | DMT family transporter [*Burkholderia* sp. A1] | 92.3 | 1.60E-153 |
| E5299_01593 | HlyC/CorC family transporter [*Burkholderia*] | 96 | 2.40E-229 |
| E5299_01786 | MFS transporter [*Burkholderia gladioli*] | 95.6 | 6.10E-206 |
| E5299_02237 | MFS transporter [*Burkholderia glumae*] | 91.4 | 1.20E-196 |
| E5299_01247 | MFS transporter [*Burkholderia*] | 93 | 7.40E-190 |
| E5299_01464 | MFS transporter [*Rhizobiales* bacterium] | 99.3 | 1.30E-246 |
| E5299_01675 | potassium transporter [*Burkholderia gladioli*] | 95.3 | 0.00E+00 |
| E5299_00922 | potassium transporter Kup [*Burkholderia* sp. A1] | 95.1 | 0.00E+00 |
| E5299_00283 | ProQ activator of osmoprotectant transporter prop [*Burkholderia gladioli*] | 70.1 | 7.70E-55 |
| E5299_01055 | PTS fructose-like transporter subunit IIB [*Burkholderia gladioli*] | 85.3 | 2.40E-243 |
| E5299_02012 | RND transporter [*Burkholderia gladioli*] | 88.2 | 0.00E+00 |
| E5299_02025 | DHA2 family efflux MFS transporter permease subunit [*Burkholderia*] | 94.5 | 9.30E-276 |
| E5299_00711 | efflux RND transporter periplasmic adaptor subunit [*Burkholderia gladioli*] | 92.3 | 7.20E-186 |
| E5299_00712 | efflux RND transporter permease subunit [*Burkholderia gladioli*] | 96.3 | 0.00E+00 |
| E5299_02527 | efflux transporter outer membrane subunit [*Burkholderia gladioli*] | 82.9 | 2.10E-227 |
| E5299_00714 | efflux transporter outer membrane subunit [*Burkholderia gladioli*] | 83 | 1.40E-209 |
| E5299_01126 | HlyD family efflux transporter periplasmic adaptor subunit [*Rhodanobacter* sp.] | 48 | 1.90E-98 |
| E5299_00013 | MATE family efflux transporter LgaH [*Burkholderia gladioli*] | 100 | 6.40E-241 |
| E5299_02375 | cyclic peptide export ABC transporter [*Burkholderia gladioli*] | 87.2 | 8.90E-280 |
| E5299_00716 | LPS export ABC transporter ATP-binding protein [*Burkholderia gladioli*] | 96.1 | 5.60E-133 |
| E5299_00718 | LPS export ABC transporter periplasmic protein LptC [*Burkholderia gladioli*] | 94.5 | 3.70E-100 |
| E5299_00476 | LPS export ABC transporter permease LptF [*Burkholderia gladioli*] | 92.8 | 8.40E-181 |
| E5299_00475 | LPS export ABC transporter permease LptG [*Burkholderia*] | 93.2 | 3.30E-198 |
